# Protective effectiveness of previous infection against subsequent SARS-Cov-2 infection: systematic review and meta-analysis

**DOI:** 10.3389/fpubh.2024.1353415

**Published:** 2024-06-20

**Authors:** Wei-Hua Hu, Huan-Le Cai, Huan-Chang Yan, Han Wang, Hui-Min Sun, Yong-Yue Wei, Yuan-Tao Hao

**Affiliations:** ^1^Department of Epidemiology and Biostatistics, School of Public Health, Peking University, Beijing, China; ^2^Peking University Center for Public Health and Epidemic Preparedness and Response, Peking University, Beijing, China; ^3^Key Laboratory of Epidemiology of Major Diseases (Peking University), Ministry of Education, Beijing, China; ^4^Department of Medical Statistics, School of Public Health, Sun Yat-sen University, Guangzhou, China

**Keywords:** SARS-CoV-2, variant, naturally infection, reinfection, protective effectiveness

## Abstract

**Background:**

The protective effectiveness provided by naturally acquired immunity against SARS-CoV-2 reinfection remain controversial.

**Objective:**

To systematically evaluate the protective effect of natural immunity against subsequent SARS-CoV-2 infection with different variants.

**Methods:**

We searched for related studies published in seven databases before March 5, 2023. Eligible studies included in the analysis reported the risk of subsequent infection for groups with or without a prior SARS-CoV-2 infection. The primary outcome was the overall pooled incidence rate ratio (*IRR*) of SARS-CoV-2 reinfection/infection between the two groups. We also focused on the protective effectiveness of natural immunity against reinfection/infection with different SARS-CoV-2 variants. We used a random-effects model to pool the data, and obtained the bias-adjusted results using the trim-and-fill method. Meta-regression and subgroup analyses were conducted to explore the sources of heterogeneity. Sensitivity analysis was performed by excluding included studies one by one to evaluate the stability of the results.

**Results:**

We identified 40 eligible articles including more than 20 million individuals without the history of SARS-CoV-2 vaccination. The bias-adjusted efficacy of naturally acquired antibodies against reinfection was estimated at 65% (pooled *IRR* = 0.35, 95% CI = 0.26–0.47), with higher efficacy against symptomatic COVID-19 cases (pooled *IRR* = 0.15, 95% CI = 0.08–0.26) than asymptomatic infection (pooled *IRR* = 0.40, 95% CI = 0.29–0.54). Meta-regression revealed that SARS-CoV-2 variant was a statistically significant effect modifier, which explaining 46.40% of the variation in *IRR*s. For different SARS-CoV-2 variant, the pooled *IRRs* for the Alpha (pooled *IRR* = 0.11, 95% CI = 0.06–0.19), Delta (pooled *IRR* = 0.19, 95% CI = 0.15–0.24) and Omicron (pooled *IRR* = 0.61, 95% CI = 0.42–0.87) variant were higher and higher. In other subgroup analyses, the pooled *IRR*s of SARS-CoV-2 infection were statistically various in different countries, publication year and the inclusion end time of population, with a significant difference (*p* = 0.02, *p* < 0.010 and *p* < 0.010), respectively. The risk of subsequent infection in the seropositive population appeared to increase slowly over time. Despite the heterogeneity in included studies, sensitivity analyses showed stable results.

**Conclusion:**

Previous SARS-CoV-2 infection provides protection against pre-omicron reinfection, but less against omicron. Ongoing viral mutation requires attention and prevention strategies, such as vaccine catch-up, in conjunction with multiple factors.

## Highlights

- The efficacy of naturally immunity against reinfection was estimated at 65% (*IRR* = 0.35, 95% confidence interval (CI) = 0.26–0.47).
- For different SARS-CoV-2 variant, the pooled *IRRs* for the Alpha (*IRR* = 0.11), Delta (*IRR* = 0.19) and Omicron (*IRR* = 0.61) variant means a progressively lower protective effectiveness.

## Introduction

SARS-CoV-2 has evolved into many variants since its initial outbreak in 2019, and the WHO has identified the Alpha, Beta, Gamma, Delta, and Omicron variants as variations of concern (VOCs). The Beta and Delta variants are distinguished by specific combinations of unique mutations, which can potentially lead to structural and functional abnormalities (1). Studies have demonstrated that these variants are associated with a higher risk compared to the Alpha and Gamma variants, as shown by a higher hospitalization rate, severity of illness, and mortality (2). Moreover, the Omicron variant emerged in late November 2021 and possesses a significantly higher number of mutations in the Spike protein compared to the afore-mentioned VOCs, surpassing them by 3–4 times (3). Consequently, the highly contagious Omicron variant quickly became the dominant strain and widespread around the world (4, 5). This, in conjunction with the gradual relaxation of strict COVID-19 control measures, led to a SARS-CoV-2 infection peak at the end of 2022 (6).

To date, the vast majority of the world’s population has been infected with SARS-CoV-2 at least once, and the issue of reinfection has become a concern. Although most people have received a COVID-19 booster vaccination, the ability of vaccines to protect against infection of Omicron is still controversial due to its great number of mutations in the spike protein, which led to antigen escape (7). Besides, studies have shown that the neutralization titer induced by previous vaccination would drop significantly after 6 months of vaccination (8) and could not be detected after 1 year (9). In such cases, the immunity built up after natural infection may be a key aspect to fight against reinfection.

With the emergence of new variants of SARS-CoV-2, there has been a significant increase in reinfection rates. For example, a meta-analysis revealed an overall reinfection rate of 0.97% [95% confidence interval (CI) = 0.71–1.27%]. However, studies providing specific data on the Alpha wave showed a reinfection rate of 0.57% (95% CI: 0.28–0.94%), which rose to 1.25% (95% CI: 0.97–1.55%) with the Delta strain, and peaked to 3.31% (95% CI: 1.15–6.53%) during the first 3 months of the Omicron wave (10). These findings suggest that the Omicron variant has a strong ability to evade immunity from previous infections (11). Correspondingly, the protection of the immunity acquired by natural infection against reinfection gradually declined with the evolution of the variants. Studies have indicated an estimated protective effect of over 82% against Alpha, Beta, and Delta variants reinfection (12, 13), whereas the protection against reinfection of the Omicron variant from previous infection was significantly reduced to 45.3%. Moreover, it will continue to decline over time (12, 14), which would last for about 5–12 months (15).

The objective of this meta-analysis was to systematically evaluate the protective effect of natural immunity against SARS-CoV-2 reinfection (both symptomatic and asymptomatic) and its trend over time. We also conducted subgroup analysis to explore divergences of natural immunity in different variants, study population, and age groups. Compared with previous relevant studies, the present study included the most recent studies up to March 5, 2023, and in particular included more studies on Omicron; and evaluated evidence from cohort studies that included only unvaccinated populations to focus on the impact of natural immunity.

## Methods

### Study strategy

We systematically searched for the relevant literature published before 5 Mar 2023 in seven databases, including four peer-reviewed databases (PubMed, Embase, Web of Science and Scopus) and three preprint platforms (medRxiv, bioRxiv, and Europe PMC). Key search terms included the following: SARS-CoV-2, natural infection, protection and reinfection. The full search strategy was described in Supplementary Table S1. A secondary reference search on all eligible studies and relevant review articles was also conducted (10, 13, 16–21). We used EndNote X8.2 (Thomson Research Soft, Stanford, CA, United States) to manage records, screen, and exclude duplicates. This study was followed the Preferred Reporting Items for Systematic Reviews and Meta-Analyses (PRISMA, Supplementary Table S2) (22), and had been registered at PROSPERO (Registration number: CRD42023405080).

### Selection criteria

Inclusion and exclusion criteria were shown in Table 1. All retrieved publications were independently assessed by two investigators according to the below criteria, and any inconsistencies were resolved by agreement in consultation with a third investigator.

**Table 1:** Inclusion and exclusion criteria for this systematic review and meta-analysis about protective effectiveness of previous infection against subsequent SARS-COV-2 infection in the world from 2020 to 2022.

Characteristic	Inclusion criteria	Exclusion criteria
Study type	1. Cohort study	–
Participants	2. Population without a history of COVID-19 vaccination	–
Sample size	3. ≥10 participants in each group	–
SARS-CoV-2 serology testing at baseline	4. Done	–
Confirmation of COVID-19 cases during follow-up	5. Nucleic acid testing or antigenic rapid diagnostic tests	–
Data reported	6. The study must have compared the risk of SARS-CoV-2 reinfection/infection between baseline seropositive and seronegative groups	1. The study only used odds ratio as an effect size indicator and did not report original data

### Data extraction and quality assessment

A standardized electronic data collection form will be used to extract the following data from included studies: (1) literature information (i.g., study title, first author, title, publication or preprint date), (2) study details (e.g., study location, study population, demographic characteristics of the study population, SARS-CoV-2 variant, sample sizes, the date of study start and end, follow up time, effect measure, the type of target antibodies, the reinfection/infection cases in baseline seropositive or seronegative groups, the definition of reinfection, whether researchers attempted to adjust for any potential covariates, *IRRs* and 95% CI). We calculated the *IRR* by constructing a 2 × 2 contingency table for those study in which the *IRR* was not reported directly. We used the Newcastle–Ottawa quality assessment scale to evaluate the risk of bias of the included cohort studies. A score of 0–3 stars was considered a low-quality study, a score of 4–6 stars was considered a moderate-quality study, and a score of 7–9 stars was considered a high-quality study. Data extraction and quality assessment was conducted independently by two investigators and checked by a third investigator, and disagreements were resolved through discussion.

### Statistical analysis

We performed a meta-analysis to estimate the pooled incidence rate ratio (*IRR*) and its 95% CI for estimating the risk of subsequent infection between the baseline seropositive and seronegative groups. The primary outcome was the risk of SARS-CoV-2 reinfection/infection between the two groups, while the second outcome was the risk of symptomatic and asymptomatic SARS-CoV-2 reinfection/infection between the two groups. A suitable model (Fixed-effects or random-effects model) was used to pool the rates across studies separately, based on the heterogeneity between estimates which was evaluated by using the *I*-squared (*I*^2^) (23). Fixed-effects models would be used if *I*^2^ ≤ 50%, which represents low to moderate heterogeneity, and random-effects models would be used if *I*^2^ ≥ 50%, representing substantial heterogeneity. We performed meta-regression to explore between-study heterogeneity.

Subgroup analyses of the primary outcome were performed in the following groups: SARS-CoV-2 variant (Alpha, Delta, and Omicron), definition of reinfection (two positive SARS-CoV-2 PCR test results at least 60 or 90 days apart), population (HCWs or general population), age (<60 years old or ≥60 years old, <55 years old or ≥55 years old), country, publication year (2020, 2021, or 2022), inclusion end time of population (every 6 months from 2020 to 2022), and study quality (moderate or high). The classification criteria for each subgroup are described in the Supplementary Table S3. Bubble plots were used to explore trends in the immune protection acquired from natural infection with COVID-19. We used funnel plots and Begg’s test to examine the potential for publication bias. If the results are suggestive of publication bias, we will further provide bias-adjusted results using trim-and-fill, a non-parametric method based on examining the funnel plot’s asymmetry. We conducted sensitivity analysis with the one-study-at-a-time method adopted for assessing the reliability of the results. All statistical analyses were conducted using meta libraries in R 4.0.5.

## Results

**Figure 1:**
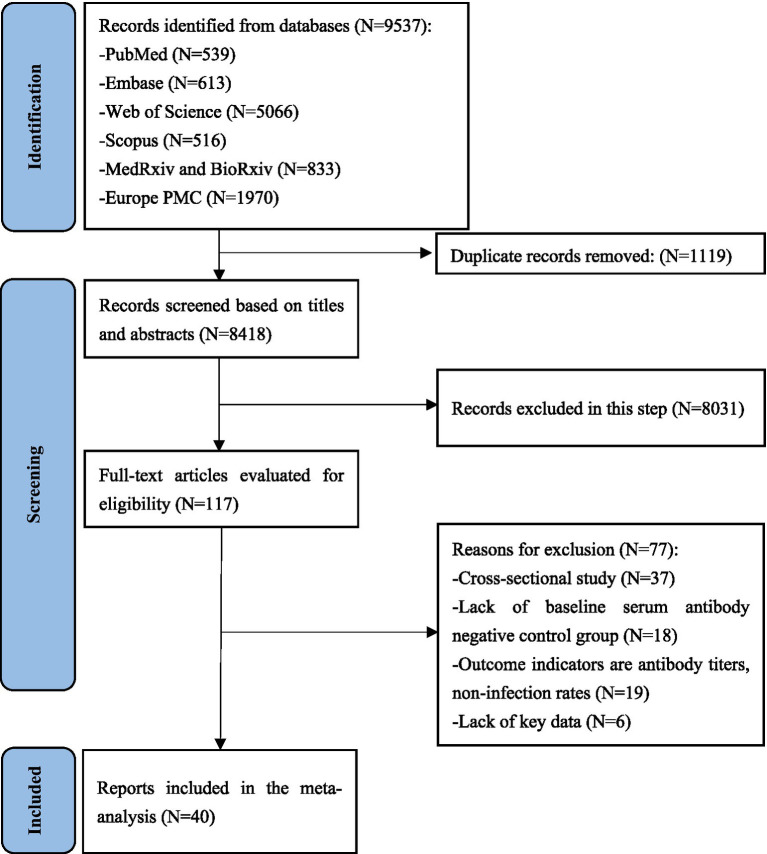
PRISMA flow diagram for this systematic review and meta-analysis about protective effectiveness of previous infection against subsequent SARS-COV-2 infection in the world from 2020 to 2022.

A total of 9,537 relevant records were identified, of which 1,119 duplicate records were removed. Eight thousand, four hundred eighteen article titles and abstracts were screened and 117 underwent full-text review. Finally, 40 unique articles reporting data for 52 studies were included in this meta-analysis (Figure 1). After a secondary reference search of all eligible studies and relevant review articles, no new studies were included. The 40 eligible articles included more than 20 million COVID-19 unvaccinated individuals without the history of COVID-19 vaccination. The sample sizes of the included studies ranged from 209 to 8,901,064 (median: 15075). Among the 40 unique articles, 11 studies were conducted in the United States, 9 in the United Kingdom, four in Switzerland, three in Qatar, two each in Sweden, Nicaragua, Italy and Israel, and one each in Austria, Bangladesh, Denmark, France and India. The mean/median ages of the enrolled participants were mostly less than 60 years old, with only two studies reporting median age over 60 years old. The study populations mainly included the general population, HCWs, care home residents and staffs, and hemodialysis patients. The included studies initiated between January 2020 and September 2021, and the length of the follow-up time ranged from 1.47 to 24.07 months. Different studies have used different window periods between positive PCR tests and baseline seropositive or previous RNA-positive results in defining reinfection. This is due to the fact that most studies were initiated in the early stages of the COVID-19 pandemic, when the persistence of SARS-CoV-2 RNA was not clearly understood. Of the included studies, 23 defined reinfection as two positive SARS-CoV-2 PCR test results at least 90 days apart, and 4 defined reinfection as two positive SARS-CoV-2 PCR test results at least 60 days apart, 1 study each defined reinfection as two positive SARS-CoV-2 PCR tests separated by a period of 270 or 28 days, and the remainder of the studies did not report a specific definition of reinfection. The quality score of study according to the NOS ranged from 4 to 9, with 14 studies of high quality, 26 studies of moderate quality, and none of low quality (Supplementary Table S4). The main characteristics of 40 eligible studies were summarized in Table 2.

**Table 2:** Description of included studies in this systematic review and meta-analysis about protective effectiveness of previous infection against subsequent SARS-COV-2 infection in the world from 2020 to 2022.

ID	Authors, year	Location	Population	Sample size	Median/mean age	Variant	Study start time	Study end time	Length of follow-up (months)	Effect measure	Quality assessment
1	Maier et al. (24)	Nicaragua	General population	2,123	–	Gamma and Delta	2020-03-01	2021-10-14	4.10	Adjusted RR	6 (MQ)
2	Lumley et al. (25)	United Kingdom	HCWs	13,109	39	Alpha	2020-04-23	2021-02-28	4.10	Adjusted RR	5 (MQ)
3	Jeffery-Smith et al. (26)	United Kingdom	Care home residents and staffs	209	84	–	2020-05-01	2020-10-31	8.97	RR	5 (MQ)
4	Lumley et al. (27)	United Kingdom	HCWs	12,541	38	–	2020-04-23	2020-11-30	9.87	Adjusted RR	5 (MQ)
5	Hansen et al. (28)	Denmark	General population	525,339	–	–	2020-02-26	2020-12-31	9.90	Adjusted RR	7 (HQ)
6	Harvey et al. (29)	United States	General population	3,257,478	48	–	2020-01-08	2020-08-26	10.77	RR	6 (MQ)
7	Kim et al. (30)	United States	General population	325,157	48.8	Delta	2020-03-09	2021-09-09	11.50	RR	7 (HQ)
7	Kim et al. (30)	United States	General population	152,656	48.8	–	2020-03-09	2021-09-09	5.83	RR	7 (HQ)
8	Kohler et al. (31)	Switzerland	HCWs	4,812	38.9	–	2020-06-22	2021-03-09	6.13	RR	5 (MQ)
9	Krutikov et al. (32)	United Kingdom	Care home residents and staffs	682	86	–	2020-10-01	2021-02-01	21.37	Adjusted RR	6 (MQ)
9	Krutikov et al. (32)	United Kingdom	Care home residents and staffs	1,429	47	–	2020-10-01	2021-02-01	21.37	Adjusted RR	6 (MQ)
10	Leidi (33)	Switzerland	Essential workers	10,457	44	–	2020-05-01	2021-01-25	1.47	Adjusted HR	5 (MQ)
11	Jeffery-Smith et al. (34)	United Kingdom	Care home residents and staffs	1,377	Care home residents: 87, Staffs: 49	Alpha	2020–04–10	2021-01-31	4.20	Adjusted RR	6 (MQ)
12	Leidi et al. (35)	Switzerland	General population	8,344	47	–	2020-04-03	2021-01-25	6.07	HR	6 (MQ)
13	Havervall et al. (36)	Sweden	HCWs and patients	1935	46	–	2020-04-09	2021-02-26	8.63	RR	4 (MQ)
14	Hall et al. (37)	United Kingdom	HCWs	25,661	46	–	2020-02-01	2021-01-11	6.27	Adjusted RR	7 (HQ)
15	Letizia et al. (38)	United States	Marine recruits	3,249	19	–	2020-05-11	2020-11-02	6.03	Adjusted HR	6 (MQ)
16	Cohen et al. (39)	United States	Hemodialysis patients	2,337	59.5	–	2020-07-01	2021-01-01	13.17	Adjusted RR	7 (HQ)
17	Chemaitelly et al. (40)	Qatar	General population	581,276	32	–	2020-02-28	2021-11-30	16.63	Adjusted HR	9 (HQ)
17	Chemaitelly et al. (40)	Qatar	General population	240,966	27	Omicron	2020-02-28	2021-11-30	9.17	Adjusted HR	9 (HQ)
18	Abu-Raddad et al. (41)	Qatar	General population	291,309	34	Alpha	2021-01-18	2021-03-03	8.60	RR	7 (HQ)
19	Schuler et al. (42)	United States	HCWs or patients	338	41	–	–	–	5.50	RR	5 (MQ)
20	Dimeglio et al. (43)	France	HCWs	8,758	–	–	2020-06-10	2020-12-09	5.50	–	5 (MQ)
21	Abu-Raddad et al. (44)	Qatar	General population	192,984	35, 38	–	2020–04–16	2020-12-31	5.50	HR	8 (HQ)
22	Abo-Leyah et al. (45)	United Kingdom	HCWs	2063	46	–	2020-05-28	2020-12-02	5.50	Adjusted HR	6 (MQ)
23	Vitale et al. (46)	Italy	General population	15,075	59	–	2020-02-01	2020-07-31	5.50	Adjusted RR	7 (HQ)
24	Maier et al. (47)	Nicaragua	General population	2,338	24	–	2020-03-01	2021-03-31	5.50	RR	7 (HQ)
25	Rahman et al. (48)	Bangladesh	HCWs	1,644	38.4	–	2020-03-19	2021-07-31	7.60	RR	6 (MQ)
26	Shields et al. (49)	United Kingdom	HCWs	1,507	37	–	2020-05-01	2021-01-31	24.07	Adjusted RR	6 (MQ)
27	Mishra et al. (50)	India	General population	2,238	–	–	–	–	10.00	RR	5 (MQ)
28	Patalon et al. (51)	Israel	General population	458,959	–	Delta	2021-07-01	2021-12-13	18.77	Adjusted RR	7 (HQ)
28	Patalon et al. (51)	Israel	General population	458,959	–	Delta	2021-07-01	2021-12-13	3.27	Adjusted RR	7 (HQ)
28	Patalon et al. (51)	Israel	General population	458,959	–	Delta	2021-07-01	2021-12-13	9.03	Adjusted RR	7 (HQ)
28	Patalon et al. (51)	Israel	General population	458,959	–	Delta	2021-07-01	2021-12-13	10.10	Adjusted RR	7 (HQ)
28	Patalon et al. (51)	Israel	General population	458,959	–	Delta	2021-07-01	2021-12-13	7.57	Adjusted RR	7 (HQ)
28	Patalon et al. (51)	Israel	General population	458,959	–	Delta	2021-07-01	2021-12-13	4.10	Adjusted RR	7 (HQ)
29	Muir et al. (52)	United Kingdom	Hemodialysis patients	217	pos: 54.4, neg: 53.6	–	2020-05-30	2021-01-15	4.10	RR	6 (MQ)
30	Rothberg et al. (53)	United States	General population	635,341	47.3	Omicron	2020-03-09	2022-03-01	8.97	Adjusted RR	8 (HQ)
31	Spicer et al. (54)	United States	General population	360,314	–	–	2020-03-06	2020-12-31	9.87	Adjusted RR	6 (MQ)
32	Nordstrom et al. (55)	Sweden	General population	2,039,106	39.2	–	2020-03-20	2021-10-04	9.90	Adjusted RR	9 (HQ)
33	Rennert and McMahan (56)	United States	University student	16,101	20.3	–	2020-08-19	2020-11-25	10.77	Adjusted RR	5 (MQ)
34	Manica et al. (57)	Italy	General population	6,074	50	–	2020-05-05	2021-01-31	11.50	RR	8 (HQ)
35	Pilz et al. (58)	Austria	General population	8,901,064	–	–	2020-02-01	2020-11-30	5.83	RR	6 (MQ)
36	Wilkins et al. (59)	United States	HCWs	6,510	41	–	2020-05-26	2021-01-08	6.13	Adjusted RR	6 (MQ)
37	Babouee Flury et al. (60)	Switzerland	HCWs	330	36.8	Delta	2021-09-20	2022-03-06	5.5	Adjusted RR	6 (MQ)
37	Babouee Flury et al. (60)	Switzerland	HCWs	330	36.8	Omicron	2021-09-20	2022-03-06	5.5	Adjusted RR	6 (MQ)
38	Kim et al. (30)	United States	General population	325,157	50.1	Delta	2020-12-31	2021-09-09	8.3	RR	6 (MQ)
38	Kim et al. (30)	United States	General population	152,656	52.6	Delta	2020-08-30	2021-09-09	12.3	RR	6 (MQ)
39	Patalon et al. (61)	Israel	Adolescents	458,959	66.3% 5-11 years	Delta	2021-07-01	2021-12-13	5.4	Adjusted RR	7 (HQ)
40	Rothberg et al. (53)	United States	General population	362,800	50.6	Omicron	2020-03-09	2022-03-01	23.7	RR	6 (MQ)
40	Rothberg et al. (53)	United States	General population	104,856	51.4	Omicron	2021-03-29	2022-03-01	11	RR	6 (MQ)
40	Rothberg et al. (53)	United States	General population	98,605	43.6	Omicron	2021-06-28	2022-03-01	8	RR	6 (MQ)

**Figure 2:**
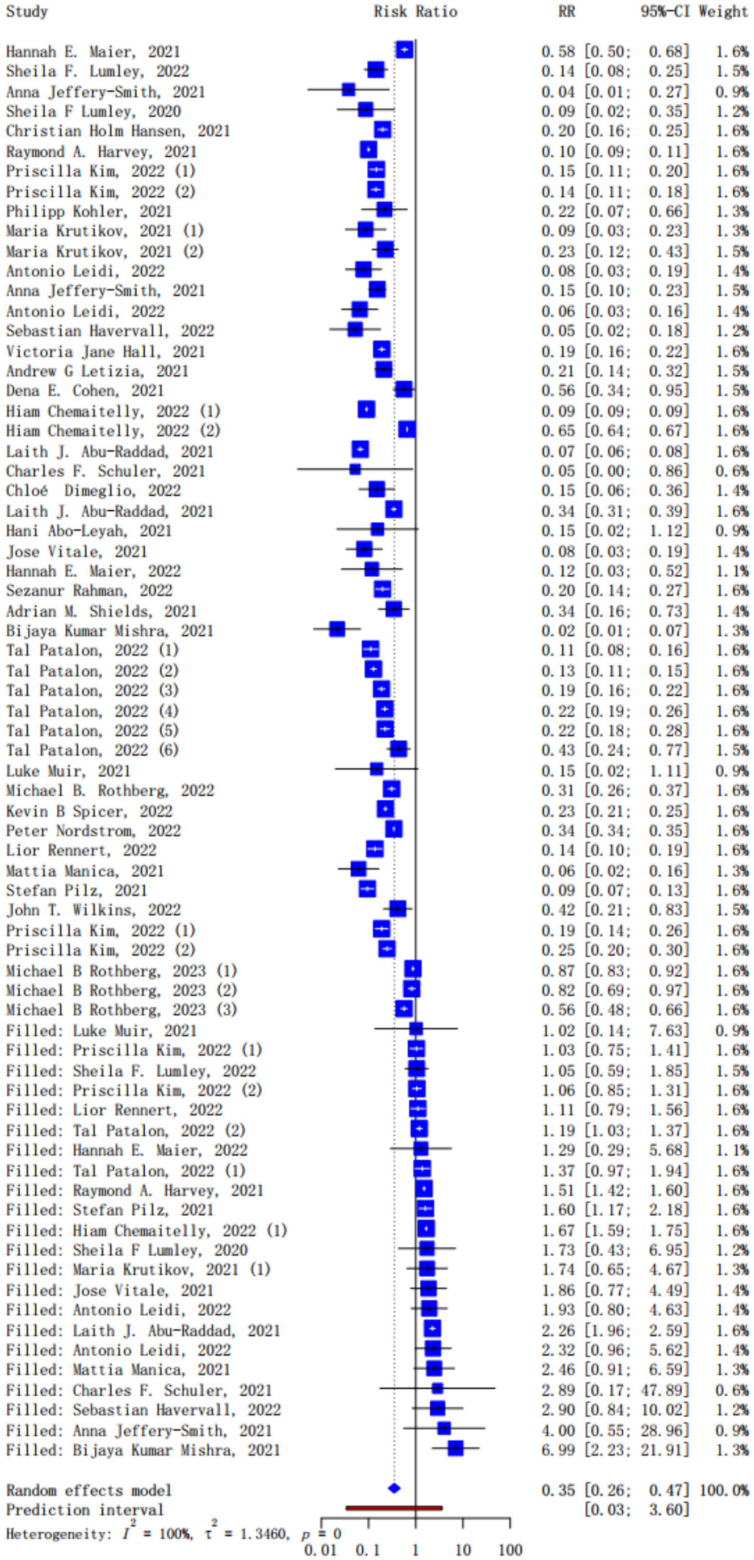
Forest plot of the pooled incidence rate ratio for SARS-CoV-2 infection comparing baseline seropositive and seronegative individuals (trim-and-fill method).

The asymmetry in funnel plot and the result of Begg’s test suggested a possible publication bias in the included studies (*p* < 0.05), so we adopted the trim-and-fill method. The funnel plot for publication bias before and after trimming and filling were shown in Supplementary Figure S3. The pooled results for the protection of naturally acquired antibodies against future SARS-CoV-2 infection after using the trim-and-fill method were shown in Figure 2, while the original results without the trim-and-fill method were shown in Supplementary Figure S1. Adopting random effect meta-analysis models, we observed significant protection against SARS-CoV-2 reinfection in the seropositive population compared with seronegative individuals (pooled *IRR* = 0.35, 95% CI = 0.26–0.47). The original pooled *IRRs* without the trim-and-fill method was 0.19 (95% CI = 0.15–0.23). In the sensitivity analysis for the original result, the pooled *IRRs* of remaining studies ranges from 0.15–0.24 after removing any one of the studies, which suggested the good reliability of the pooled *IRR* (Supplementary Figure S2).

**Figure 3:**
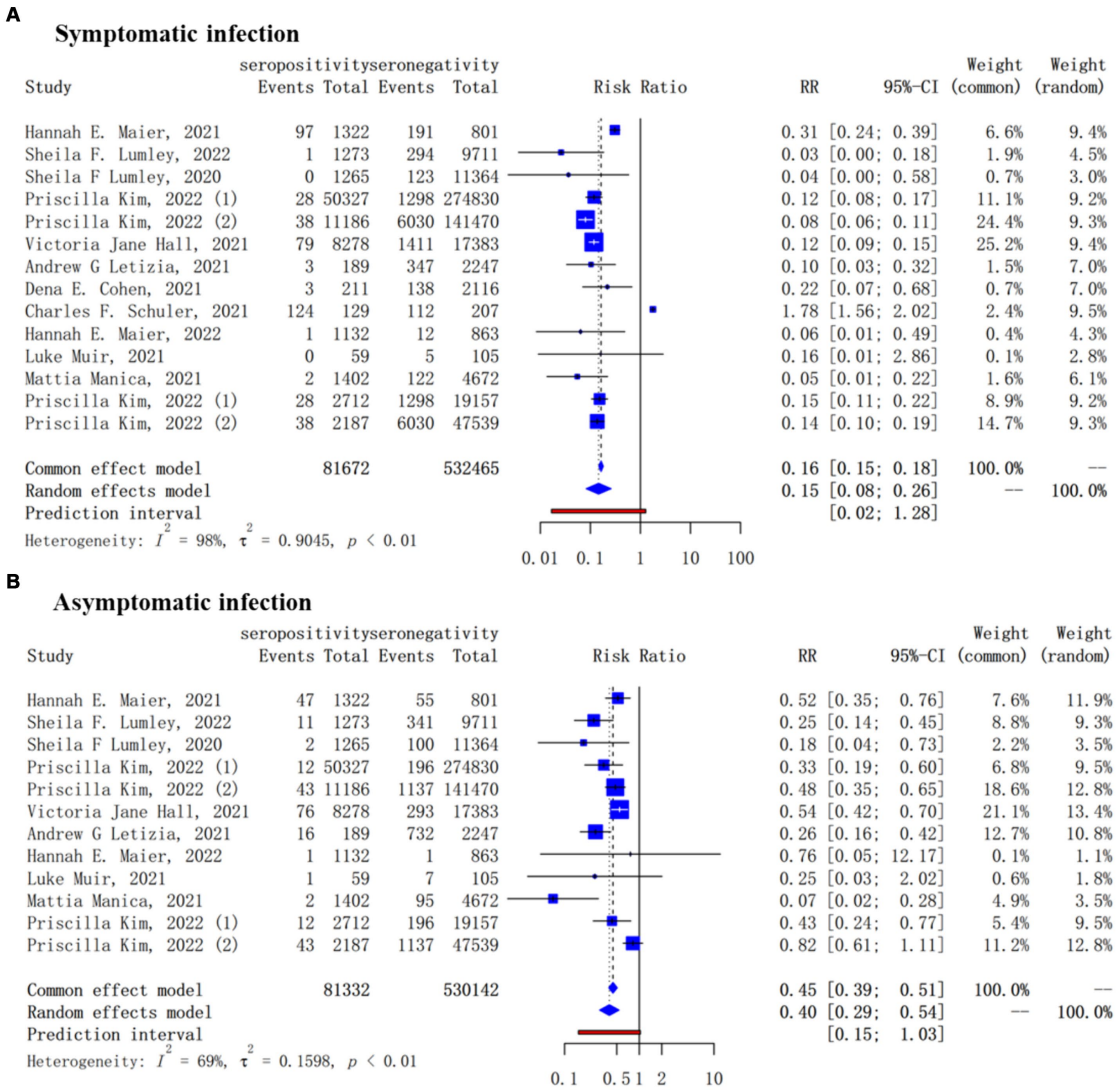
Forest plot of the protection provided by naturally acquired antibodies against future symptomatic **(A)** and asymptomatic **(B)** COVID-19 between baseline seropositive and seronegative individuals.

For secondary outcome, 12 studies reported the protection of the antibodies induced by a previous infection against future symptomatic between baseline seropositive and seronegative groups while there were 10 studies for asymptomatic reinfections. Natural infections of SARS-CoV-2 provided a lower level of protection against asymptomatic infection (pooled IRR = 0.40, 95% CI = 0.29–0.54) than symptomatic COVID-19 cases (pooled *IRR* = 0.15, 95% CI = 0.08–0.26) (Figure 3).

**Figure 4:**
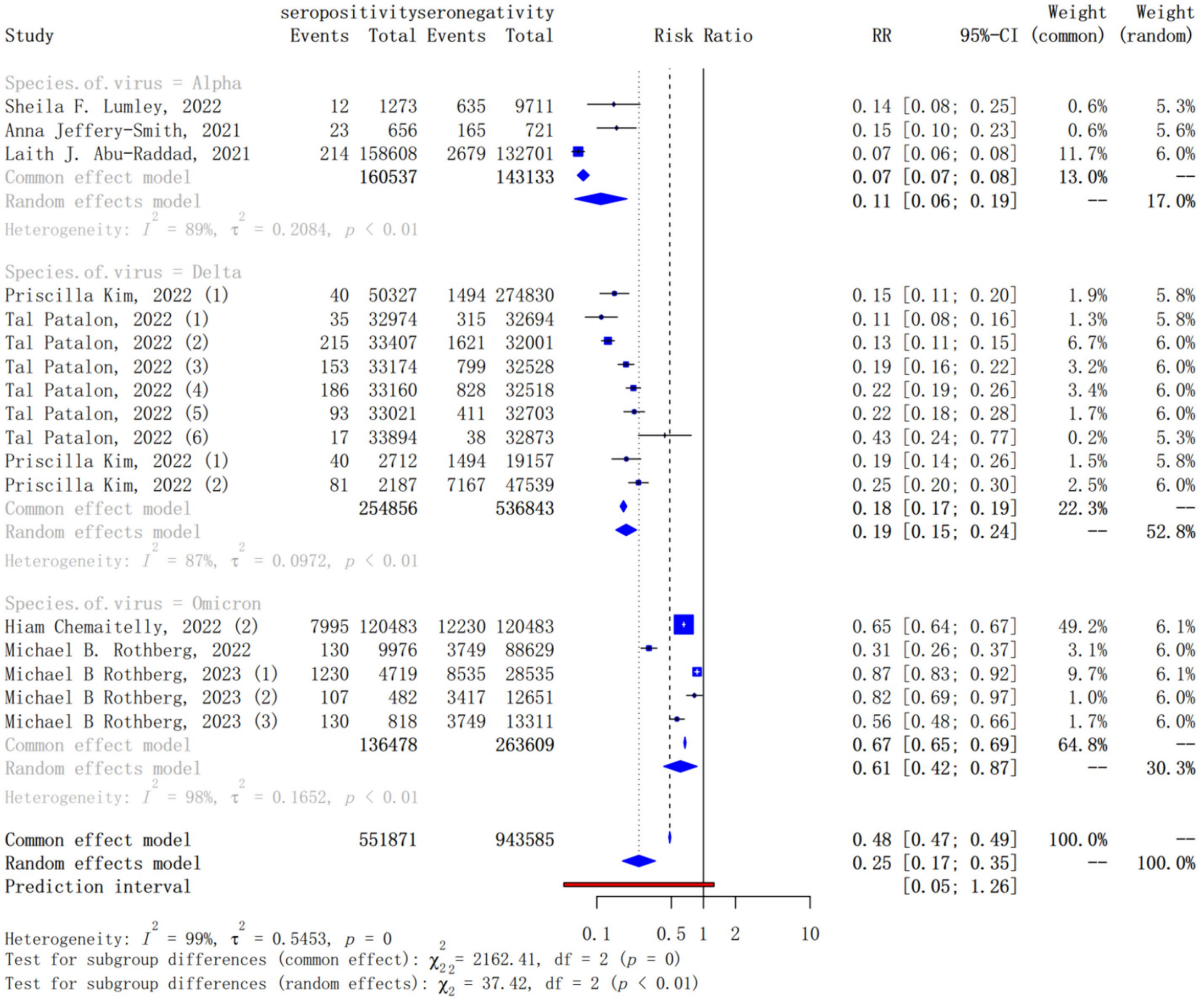
Forest plot of the pooled incidence rate ratio for different SARS-CoV-2 variant infection comparing baseline seropositive with seronegative individuals.

Meta-regression revealed that SARS-CoV-2 variant was a statistically significant effect modifier, which explaining 46.40% of the variation in *IRR*s. The subgroup analysis for different SARS-CoV-2 variant showed that the pooled *IRRs* for the Alpha (pooled *IRR* = 0.11, 95% CI = 0.06–0.19), Delta (pooled *IRR* = 0.19, 95% CI = 0.15–0.24) and Omicron (pooled *IRR* = 0.61, 95% CI = 0.42–0.87) variant were higher and higher, that is, the protection of natural infection for reinfection against these variants was progressively lower (Figure 4).

In other subgroup analyses, statistically significant differences were observed in the subgroup analysis of the country (the pooled *IRR* = 0.20, 95% CI = 0.16–0.25, *p* = 0.02, Supplementary Figure S6), the publication year (the pooled *IRR* = 0.19, 95% CI = 0.15–0.23, *p* < 0.010, Supplementary Figure S8–1) and the inclusion end time of population (the pooled *IRR* = 0.20, 95% CI = 0.16–0.24, *p* < 0.010, Supplementary Figure S8–2). In different countries, Nicaragua was found a lower level of protection against reinfection (pooled *IRR* = 0.31, 95% CI = 0.07–1.43), while Italy was found a higher level of protection against reinfection (pooled *IRR* = 0.07, 95% CI = 0.04–0.14). For studies published from 2020 to 2023, the pooled *IRR* was on the rise. It is 0.09 (95% CI = 0.02–0.35) for studies published in 2020, 0.15 (95% CI = 0.11–0.22) in 2021, 0.19 (95% CI = 0.15–0.23) in 2022 and 0.74 (95% CI = 0.57–0.97) in 2023. However, no significant differences were observed in the subgroup analysis of the definition of reinfection (the pooled *IRR* = 0.21, 95% CI = 0.17–0.27, *p* = 0.06, Supplementary Figure S10), the population type (the pooled *IRR* = 0.20, 95% CI = 0.16–0.26, *p* = 0.40, Supplementary Figure S4) and the study quality score (the pooled *IRR* = 0.19, 95% CI = 0.15–0.23, *p* = 0.82, Supplementary Figure S7). In addition, the pooled *IRRs* of reinfection was higher in participants aged less than 60 years than those greater than 60 years (0.19, 95% CI = 0.15–0.25 vs. 0.07, 95% CI = 0.03–0.18), differences (*p* < 0.04) between the two age groups were significant (Supplementary Figure S5–1). However, given that there were only two studies with a median age of over 60, the results may not be representative. Therefore, we also used the median age of 55 years as the basis of grouping for exploratory analysis. We found the difference of the pooled *IRRs* in participants aged less than 55 years than those greater than 55 years (0.19, 95% CI = 0.14–0.25 vs. 0.13, 95% CI = 0.04–0.42) was not statistical (Supplementary Figure S5–2).

Most studies that reported the mean/median follow-up times were included in the bubble plot to explore the changing trends of the protection provided by naturally acquired antibodies after a prior COVID-19 infection, the protection appeared to decrease slowly over time (Supplementary Figure S9).

## Discussion

This systematic review and meta-analysis, including 40 studies and over 20 million unvaccinated individuals, provides a synthesis of the evidence that natural immunity from primary infection can prevent SARS-CoV-2 reinfection (*IRR* = 0.35), especially symptomatic reinfection (*IRR* = 0.15). Meanwhile, the protective efficacy declined during Omicron wave and varied by study location and publication year. These findings suggests that people after primary infection should still be vaccinated and use personal protections to reduce the risk of reinfection.

A high protective efficacy of natural infection against SARS-CoV-2 reinfection has been reported in the available systematic reviews (10, 13, 62–64), but our estimate (65%) is much lower than others (>80%). On one side, the original estimated efficacy in our primary analysis was 81% (Supplementary Figure S2) and in line with the previous estimates, but the conservative estimate was obtained with a non-parametric “trim-and-fill” method to reduce publication bias (65). On the other side, evidence in South Africa suggests increased risk of SARS-CoV-2 reinfection associated with emergence of Omicron (66), and we included the most recent studies during Omicron epidemic which may lead to a lower protection effect due to the omicron’s immune escape ability. Therefore, SARS-CoV-2 reinfection should be highlighted for the further prevention strategies over time.

In our study, protection against symptomatic reinfection is substantial with an estimate corresponding with the previous reviews (18, 64), while the effect on asymptomatic reinfection (60%) was weaker than on symptomatic reinfection (85%). The findings might be biased by the inadequate detection of all asymptomatic infections in those studies based on surveillance. Nevertheless, it is similar to the SIREN (SARS-CoV-2 Immunity and Reinfection Evaluation) study with the best methods, that the protective efficacy of primary infection was 93 and 52% against symptomatic and asymptomatic reinfection, respectively (37). Also, Deng et al. (16) found reinfection cases were more likely to present with mild symptoms than primary infection ones. In contrast, the meta-analysis performed by Bowe et al. (67) showed that reinfection can further increase risks of death, hospitalization, and sequelae in the acute and post-acute phase, regardless of vaccination status. Still, strategies for reinfection prevention remains to be carefully considered and evaluated.

Furthermore, the efficacy of natural infection against reinfection by the Alpha, Delta, and Omicron variants was estimated at 89, 81, and 49%, respectively. In spite of the limited number of variant-specific studies, similar pattern was observed in the sub-group analysis for the study publication year and the inclusion end time of population, that the efficacy of natural infection was lower during the period of omicron outbreak than during pre-omicron outbreak. Our findings are identical to a previous meta-analysis (10), suggesting an increase of reinfection risk as the omicron variant emerged. The low efficacy against the omicron variant might result from its unique mutations on pre-existing antibodies (68), as well as antibody neutralization (69). Accordingly, the risk of reinfection was lower among the vaccinated population than among the unvaccinated during the omicron wave, strengthening the need of multiple dose vaccination after primary infection (10). However, in addition to focusing on the rate of reinfection with a specific variant, it is equally important to assess the prevalence of long-COVID and the overall health impact on individuals following reinfection. For instance, studies have indicated that the prevalence of long-COVID is significantly lower among individuals infected with the Omicron variant compared to those infected with previous variants such as Alpha and Delta (70). Moreover, among patients with long-COVID, it was not Omicron-infected but Alpha-infected patients who had a higher prevalence of central neurological symptoms (71). Hence, it is crucial to consider multiple factors comprehensively when developing a vaccination strategy.

Due to the unavailability of data and the complexity of the study, the present study was not focused on the protective effect of natural infection with a particular SARS-CoV-2 variant on reinfection with the same variant, but rather on the protective effect of a previously naturally infection on subsequent reinfections, and if there was a difference in its protective effect on reinfections with different variants. This review currently includes 40 relevant studies published up to March 2023 for extraction 52 study data (Table 2). Of the 17 study data that reported the type of reinfection variant, 3 data focused on the protective effect of natural infection on reinfection of Alpha variant (17.65%), 9 data focused on the protective effect of natural infection on reinfection of Delta variant (52.94%), and 5 data focused on the protective effect of natural infection on reinfection of omicron variant (29.41%). The remaining 35 data were from studies that did not report a specific reinfection variant of interest, and it is highly likely that there is a mishmash of reinfection with multiple variants. Therefore, only these 17 data focusing on reinfection with a single variant were included in the subgroup analysis of viral variants in this paper. The virus has evolved over time, and the majority of the current population is infected with Omicron. However, there is a paucity of studies on the protective effect of previous infection with Omicron on reinfection with Omicron and its subsequent variants, which has not been considered at this time in this review study, and may therefore lead to an underestimation of the overall protective effect of previous infection on reinfection. In view of this, we will continue to follow up the study and plan to update the results at an appropriate time, such as in 6 months or a year later, depending on subsequent SARS-CoV-2 infections.

Here, we found poor protective effect of prior infection against SARS-CoV-2 reinfection in Nicaragua but a higher protective effect in Italy, which may be due to the lower oxford policy stringency index in the former, that is, the looser prevention and control policy; and the higher index in the latter, meaning a stricter prevention and control policy. Distinctively, our study shows a low protective efficacy of natural infection among people over 60 years old, contrast to the previous findings (13, 55, 62). It may be because the median age of only 2 studies is greater than 60, the results obtained are not representative. However, there were four studies with a median age greater than 55 and we found there was no statistical difference in the protective effect of natural infection between people over and below 55 years old.

In China, the vaccine immunity of most people has been reduced to a very low level, and the current immunity to reinfection with SARS-CoV-2 mainly relies on the natural immunity generated during the Omicron epidemic at the end of last year. Therefore, this study is very in line with China’s current national conditions and will help provide a scientific basis for preventing re-infection in the Chinese population.

However, this study was subject to limitations. Firstly, the *I*^2^ value and Cochran’s *Q* test suggests high heterogeneity between the studies in our analyses, due to the various regions, periods and populations (72). Under this circumstance, we had to accept the existence of the heterogeneity. Therefore, we used the random effects model instead of the fixed effects model to estimate the combined effect value in our meta-analysis. The greater uncertainty brought by heterogeneity to our estimate has been reflected in the method of estimation and calculation of the confidence interval under the random effects model. To explore the sources of heterogeneity and their impact on the results, we have conducted meta-regression and subgroup analyses. The meta-regression results of this study showed that the SARS-CoV-2 variant that the studies focused on and the year of publication of the studies were important sources of high heterogeneity. As the fact that the dominant strains of SARS-CoV-2 differed from year to year, we believe that the heterogeneity among studies due to different years of publication is essentially due to the different endemic strains of SARS-CoV-2 represented behind the different years, which explaining 46.40% of the variation in *IRRs*. Therefore, this review next focused on the protective effects of natural infection with SARS-CoV-2 against reinfection with different variants through subgroup analysis, which indicated the protective effects of natural infection against reinfection Alpha to Omicron gradually decreases. Compared to the overall protective effect of natural infection against reinfection, we believe that the subgroup results of the sub-variant are of greater interest and are the highlight of this study. To evaluate the stability of the results of this review, we performed a sensitivity analysis by excluding the included literature one by one. The results showed that there was no significant change in the results of the meta-analysis of the remaining studies after excluding any of them. This suggests that the included studies had stable results despite their heterogeneity. Secondly, the estimated efficacy against asymptomatic reinfection might be underestimated, for the inadequate detection. Lastly, publication bias was detected in the included studies but we used trim-and-fill method to reduce its potential effect.

## Conclusion

Our findings indicate that individuals who have previously been infected with SARS-CoV-2 possess significant protection against reinfection from pre-omicron variants. However, when it comes to the omicron variant, the level of protection against reinfection is notably diminished. This will require continued attention to viral mutation in the future and careful consideration of strategies to prevent reinfection, such as vaccine catch-up, in conjunction with other factors, such as the reinfection rate, the prevalence of long-COVID and the overall health impact on individuals following reinfection.

## Author contributions

W-HH: Writing – review & editing, Writing – original draft, Methodology, Investigation, Formal analysis, Data curation. H-LC: Writing – original draft, Data curation. H-CY: Writing – original draft, Data curation. HW: Writing – original draft, Data curation. H-MS: Writing – original draft, Data curation. Y-YW: Writing – review & editing, Methodology. Y-TH: Writing – review & editing, Supervision, Conceptualization.
