# Supplementary material for: Protective effectiveness of previous infection against subsequent SARS-Cov-2 infection: systematic review and meta-analysis
Source: Front Public Health. 2024 Jun 20;12:1353415. doi: 10.3389/fpubh.2024.1353415 (PMC11222391; doi:10.3389/fpubh.2024.1353415)

**Supplementary File**

**Protective Effectiveness of Previous Infection Against Subsequent SARS-Cov-2 Infection:**

**Systematic Review and Meta-analysis**

**Contents**

[Supplementary Table 1. Search strategy for peer-reviewed databases and preprint platforms 4](#_Toc147749149)

[Supplementary Table 2: PRISMA 6](#_Toc147749150)

[Supplementary Table 3: The classification criteria for subgroup 8](#_Toc147749151)

[Supplementary Table 4. Quality assessment of the included studies using the Newcastle–Ottawa scale. 9](#_Toc147749152)

[Supplementary Figure 1. Raw forest plot of the pooled incidence rate ratio for SARS-CoV-2 infection comparing baseline seropositive and seronegative individuals. 15](#_Toc147749153)

[Supplementary Figure 2. Sensitivity analysis for pooled incidence rate ratio 16](#_Toc147749154)

[Supplementary Figure 3. Funnel plot for publication bias before and after trimming and filling 17](#_Toc147749155)

[Supplementary Figure 4. Forest plot of the pooled incidence rate ratio for SARS-CoV-2 infection comparing baseline seropositive with seronegative individuals in general population subgroup, HCWs subgroup and hemodialysis patient subgroup 18](#_Toc147749156)

[Supplementary Figure 5-1. Forest plot of the pooled incidence rate ratio for SARS-CoV-2 infection comparing baseline seropositive with seronegative individuals in <60 years old subgroup and ≥60 years old subgroup 19](#_Toc147749157)

[Supplementary Figure 5-2. Forest plot of the pooled incidence rate ratio for SARS-CoV-2 infection comparing baseline seropositive with seronegative individuals in <55 years old subgroup and ≥55 years old subgroup 20](#_Toc147749158)

[Supplementary Figure 6. Forest plot of the pooled incidence rate ratio for SARS-CoV-2 infection comparing baseline seropositive with seronegative individuals in subgroup of different countries 21](#_Toc147749159)

[Supplementary Figure 7. Forest plot of the pooled incidence rate ratio for SARS-CoV-2 infection comparing baseline seropositive with seronegative individuals in moderate-quality and high-quality group 22](#_Toc147749160)

[Supplementary Figure 8-1. Forest plot of the pooled incidence rate ratio for SARS-CoV-2 infection comparing baseline seropositive with seronegative individuals in subgroup of different publication year 23](#_Toc147749161)

[Supplementary Figure 8-2. Forest plot of the pooled incidence rate ratio for SARS-CoV-2 infection comparing baseline seropositive with seronegative individuals in subgroups of inclusion end time of population 24](#_Toc147749162)

[Supplementary Figure 9. The changing trend of incidence rate ratio after prior infection 25](#_Toc147749163)

[Supplementary Figure 10. Forest plot of the pooled incidence rate ratio for SARS-CoV-2 infection comparing baseline seropositive with seronegative individuals in subgroup of different definitions of reinfection 26](#_Toc147749164)

# Supplementary Table 1. Search strategy for peer-reviewed databases and preprint platforms

| **Database** | **Step** | **Search strategy** |
| --- | --- | --- |
| PubMed  (PMC) | #1 | (Natural infection[Title/Abstract]) OR (Naturally Acquired Antibody[Title/Abstract]) OR (naturally acquired immunity[Title/Abstract]) OR (natural immunity[Title/Abstract]) |
|  | #2 | (COVID-19[Title/Abstract]) OR (COVID19[Title/Abstract]) OR (Coronavirus Disease 2019[Title/Abstract]) OR (2019-nCoV[Title/Abstract]) OR (2019 Novel Coronavirus[Title/Abstract]) OR (SARS-CoV-2[Title/Abstract]) OR (SARS Coronavirus 2[Title/Abstract]) OR (severe acute respiratory syndrome coronavirus 2[Title/Abstract]) |
|  | #3 | (Protect[Title/Abstract]) OR (Protection[Title/Abstract]) OR (protective[Title/Abstract]) OR (protected[Title/Abstract]) OR (prevention[Title/Abstract]) OR (Preventive[Title/Abstract]) OR (effect[Title/Abstract]) OR (effective[Title/Abstract]) OR (Effectiveness[Title/Abstract]) OR (efficacy[Title/Abstract]) OR (preventive efficacy[Title/Abstract]) OR (risk[Title/Abstract]) |
|  | #4 | #1 AND #2 AND #3 |
| Web of science | #1 | TS= (Natural infection OR Naturally Acquired Antibody OR naturally acquired immunity OR natural immunity) |
|  | #2 | TS= (COVID-19 OR COVID19 OR Coronavirus Disease 2019 OR 2019-nCoV OR 2019 Novel Coronavirus OR SARS-CoV-2 OR SARS Coronavirus 2 OR severe acute respiratory syndrome coronavirus 2) |
|  | #3 | TS= (Protect OR Protection OR protective OR protected OR prevention OR Preventive OR effect OR effective OR Effectiveness OR efficacy OR preventive efficacy OR risk) |
|  | #4 | #1 AND #2 AND #3 |
| Embase | #1 | ‘Natural infection’:ab,ti OR ‘Naturally Acquired Antibody’:ab,ti OR ‘naturally acquired immunity’:ab,ti OR ‘natural immunity’:ab,ti |
|  | #2 | ‘COVID-19’:ab,ti OR ‘COVID19’:ab,ti OR ‘Coronavirus Disease 2019’:ab,ti OR ‘2019-nCoV’:ab,ti OR ‘2019 Novel Coronavirus’:ab,ti OR ‘SARS-CoV-2’:ab,ti OR ‘SARS Coronavirus 2’:ab,ti OR ‘severe acute respiratory syndrome coronavirus 2’:ab,ti |
|  | #3 | ‘Protect’:ab,ti OR ‘Protection’:ab,ti OR ‘protective’:ab,ti OR ‘protected’:ab,ti OR ‘prevention’:ab,ti OR ‘Preventive’:ab,ti OR ‘effect’:ab,ti OR ‘effective’:ab,ti OR ‘Effectiveness’:ab,ti OR ‘efficacy’:ab,ti OR ‘preventive efficacy’:ab,ti OR ‘risk’:ab,ti |
|  | #4 | #1 AND #2 AND #3 |
| Scope | #1 | TITLE-ABS ("Natural infection" OR "Naturally Acquired Antibody" OR "naturally acquired immunity" OR "natural immunity") |
|  | #2 | TITLE-ABS (COVID-19 OR COVID19 OR "Coronavirus Disease 2019" OR "2019-nCoV" OR "2019 Novel Coronavirus" OR "SARS-CoV-2" OR "SARS Coronavirus 2" OR "severe acute respiratory syndrome coronavirus 2") |
|  | #3 | TITLE-ABS (Protect OR Protection OR protective OR protected OR prevention OR Preventive OR effect OR effective OR Effectiveness OR efficacy OR "preventive efficacy" OR risk) |
|  | #4 | #1 AND #2 AND #3 |
| Europe PMC | #1 | ("COVID-19" OR "COVID19" OR "Coronavirus Disease 2019" OR "2019-nCoV" OR "2019 Novel Coronavirus" OR "SARS-CoV-2" OR "SARS Coronavirus 2" OR "severe acute respiratory syndrome coronavirus 2") |
|  | #2 | ("Natural infection" OR "Naturally Acquired Antibody" OR "naturally acquired immunity" OR "natural immunity") |
|  | #3 | ("Protect" OR "Protection" OR "protective" OR "protected" OR "prevention" OR "Preventive" OR "effect" OR "effective" OR "Effectiveness" OR "efficacy" OR "preventive efficacy" OR "risk") |
|  | #4 | Type: Preprints |
|  | #5 | #1 AND #2 AND #3 AND #4 |
| medRxiv, bioRxiv | #1 | (COVID-19 OR SARS-CoV-2) AND (Protect*) AND (Natural*) |

* We systematically searched for the relevant literatures published before 5 Mar 2023 in above databases.

# Supplementary Table 2: PRISMA

| **Section/topic** | **#** | **Checklist item** | **Reported on page #** |
| --- | --- | --- | --- |
| **TITLE** | | |  |
| Title | 1 | Identify the report as a systematic review, meta-analysis, or both. | Page 1, Manuscript |
| **ABSTRACT** | | |  |
| Structured summary | 2 | Provide a structured summary including, as applicable: background; objectives; data sources; study eligibility criteria, participants, and interventions; study appraisal and synthesis methods; results; limitations; conclusions and implications of key findings; systematic review registration number. | Page 2, Manuscript |
| **INTRODUCTION** | | |  |
| Rationale | 3 | Describe the rationale for the review in the context of what is already known. | Page 4-5, Manuscript |
| Objectives | 4 | Provide an explicit statement of questions being addressed with reference to participants, interventions, comparisons, outcomes, and study design (PICOS). | Page 5, Manuscript |
| **METHODS** | | |  |
| Protocol and registration | 5 | Indicate if a review protocol exists, if and where it can be accessed (e.g., Web address), and, if available, provide registration information including registration number. | Page 6, Manuscript; Supplementary Table 1 |
| Eligibility criteria | 6 | Specify study characteristics (e.g., PICOS, length of follow-up) and report characteristics (e.g., years considered, language, publication status) used as criteria for eligibility, giving rationale. | Page 6, Manuscript |
| Information sources | 7 | Describe all information sources (e.g., databases with dates of coverage, contact with study authors to identify additional studies) in the search and date last searched. | Page 5, Manuscript;  Supplementary Table 1 |
| Search | 8 | Present full electronic search strategy for at least one database, including any limits used, such that it could be repeated. | Supplementary Table 1 |
| Study selection | 9 | State the process for selecting studies (ie, screening, eligibility, included in systematic review, and, if applicable, included in the meta-analysis). | Page 6, Manuscript |
| Data collection process | 10 | Describe method of data extraction from reports (e.g., piloted forms, independently, in duplicate) and any processes for obtaining and confirming data from investigators. | Page 6, Manuscript |
| Data items | 11 | List and define all variables for which data were sought (e.g., PICOS, funding sources) and any assumptions and simplifications made. | Supplementary Table 3 |
| Risk of bias in individual studies | 12 | Describe methods used for assessing risk of bias of individual studies (including specification of whether this was done at the study or outcome level), and how this information is to be used in any data synthesis. | None |
| Summary measures | 13 | State the principal summary measures (e.g., risk ratio, difference in means). | Page 7, Manuscript |
| Synthesis of results | 14 | Describe the methods of handling data and combining results of studies, if done, including measures of consistency (e.g., I^2^) for each meta-analysis. | Page 7, Manuscript |
| Risk of bias across studies | 15 | Specify any assessment of risk of bias that may affect the cumulative evidence (e.g., publication bias, selective reporting within studies). | Page 7, Manuscript |
| Additional analyses | 16 | Describe methods of additional analyses (e.g., sensitivity or subgroup analyses, meta-regression), if done, indicating which were pre-specified. | Page 8, Manuscript |
| **RESULTS** | | |  |
| Study selection | 17 | Give numbers of studies screened, assessed for eligibility, and included in the review, with reasons for exclusions at each stage, ideally with a flow diagram. | Page 8, 22, Manuscript |
| Study characteristics | 18 | For each study, present characteristics for which data were extracted (e.g., study size, PICOS, follow-up period) and provide the citations. | Page 26-30, Manuscript；  Supplementary Table 4 |
| Risk of bias within studies | 19 | Present data on risk of bias of each study and, if available, any outcome level assessment (see item 12). | None |
| Results of individual studies | 20 | For all outcomes considered (benefits or harms), present, for each study: (a) simple summary data for each intervention group (b) effect estimates and confidence intervals, ideally with a forest plot. | Page 26-30, Manuscript；  Supplementary Table 4 |
| Synthesis of results | 21 | Present results of each meta-analysis done, including confidence intervals and measures of consistency. | Page 9-11, Manuscript |
| Risk of bias across studies | 22 | Present results of any assessment of risk of bias across studies (see Item 15). | Page 9, Manuscript |
| Additional analysis | 23 | Give results of additional analyses, if done (e.g., sensitivity or subgroup analyses, meta-regression [see Item 16]). | Page 9, Manuscript |
| **DISCUSSION** | | |  |
| Summary of evidence | 24 | Summarize the main findings including the strength of evidence for each main outcome; consider their relevance to key groups (e.g., healthcare providers, users, and policy makers). | Page 11, Manuscript |
| Limitations | 25 | Discuss limitations at study and outcome level (e.g., risk of bias), and at review-level (e.g., incomplete retrieval of identified research, reporting bias). | Page 13, Manuscript |
| Conclusions | 26 | Provide a general interpretation of the results in the context of other evidence, and implications for future research. | Page 14, Manuscript |
| **FUNDING** | | |  |
| Funding | 27 | Describe sources of funding for the systematic review and other support (e.g., supply of data); role of funders for the systematic review. | Page 15, Manuscript |

# Supplementary Table 3: The classification criteria for subgroup

| **Subgroup** | **Classification criteria** |
| --- | --- |
| **Species of virus** |  |
| Alpha | The study was focus on the reinfection/infection of SARS-CoV-2 on Alpha variant. |
| Delta | The study was focus on the reinfection/infection of SARS-CoV-2 on Delta variant. |
| Omicron | The study was focus on the reinfection/infection of SARS-CoV-2 on Omicron variant. |
| **Population** |  |
| HCWs | Health care workers include physicians, nurses, emergency medical personnel, dental professionals and students, medical and nursing students, laboratory technicians, pharmacists, hospital volunteers, and hospital administrative staff.  (https://www.cdc.gov/vaccines/adults/rec-vac/hcw.html) |
| General population | The studies included the community population and did not target a specific population. |
| **Age** |  |
| < 60 years old | The median or mean age of participants was < 60 years. |
| ≥ 60 years old | The median or mean age of participants was ≥ 60 years. |
| < 55 years old | The median or mean age of participants was < 55 years. |
| ≥ 55 years old | The median or mean age of participants was ≥ 55 years. |
| **Study quality** |  |
| Moderate | The Newcastle–Ottawa score of the study was 4–6 stars. |
| High | The Newcastle–Ottawa score of the study was 7-9 stars. |

# Supplementary Table 4. Quality assessment of the included studies using the Newcastle–Ottawa scale.

| **No.** | **Study** | **Scores** | **Selection** | | | |  | **Comparability** |  | **Outcome** | | |
| --- | --- | --- | --- | --- | --- | --- | --- | --- | --- | --- | --- | --- |
|  |  |  | Representativeness of the exposed cohort | Selection of the non-exposed cohort | Ascertainment of exposure | Demonstration that outcome of interest was not present at start of study |  | Comparability of cohorts on the basis of the design or analysis^^^ |  | Assessment of outcome | Was follow up long enough for outcomes to occur | Adequacy of follow up of cohorts |
| 1 | Hannah E. Maier et al, 2021 | 6 (MQ) | 1 | 1 | 1 | 0 |  | 1 |  | 1 | 1 | 0 |
| 2 | Sheila F. Lumley et al, 2022 | 5 (MQ) | 0 | 1 | 1 | 0 |  | 1 |  | 1 | 1 | 0 |
| 3 | Anna Jeffery-Smith et al, 2021 | 5 (MQ) | 0 | 1 | 1 | 0 |  | 1 |  | 1 | 1 | 0 |
| 4 | Sheila F Lumley et al, 2020 | 5 (MQ) | 0 | 1 | 1 | 0 |  | 1 |  | 1 | 1 | 0 |
| 5 | Christian Holm Hansen et al, 2021 | 7 (HQ) | 1 | 1 | 1 | 0 |  | 1 |  | 1 | 1 | 1 |
| 6 | Raymond A. Harvey et al, 2021 | 6 (MQ) | 1 | 1 | 1 | 0 |  | 1 |  | 1 | 1 | 0 |
| 7 | Priscilla Kim et al, 2022 | 7 (HQ) | 1 | 1 | 1 | 0 |  | 1 |  | 1 | 1 | 1 |
| 8 | Philipp Kohler et al, 2021 | 5 (MQ) | 0 | 1 | 1 | 0 |  | 1 |  | 1 | 1 | 0 |
| 9 | Maria Krutikov et al, 2021 | 6 (MQ) | 0 | 1 | 1 | 0 |  | 1 |  | 1 | 1 | 1 |
| 9 | Maria Krutikov et al, 2021 | 6 (MQ) | 0 | 1 | 1 | 0 |  | 1 |  | 1 | 1 | 1 |
| 10 | Antonio Leidi et al, 2022 | 5 (MQ) | 0 | 1 | 1 | 0 |  | 1 |  | 1 | 1 | 0 |
| 11 | Anna Jeffery-Smith et al, 2021 | 6 (MQ) | 0 | 1 | 1 | 0 |  | 1 |  | 1 | 1 | 1 |
| 12 | Antonio Leidi et al, 2022 | 6 (MQ) | 1 | 1 | 1 | 0 |  | 1 |  | 1 | 1 | 0 |
| 13 | Sebastian Havervall et al, 2022 | 4 (MQ) | 0 | 1 | 1 | 0 |  | 1 |  | 1 | 0 | 0 |
| 14 | Victoria Jane Hall et al, 2021 | 7 (HQ) | 0 | 1 | 1 | 0 |  | 2 |  | 1 | 1 | 1 |
| 15 | Andrew G Letizia et al, 2021 | 6 (MQ) | 0 | 1 | 1 | 0 |  | 1 |  | 1 | 1 | 1 |
| 16 | Dena E. Cohen et al, 2021 | 7 (HQ) | 0 | 1 | 1 | 1 |  | 1 |  | 1 | 1 | 1 |
| 17 | Hiam Chemaitelly et al, 2022 | 9 (HQ) | 1 | 1 | 1 | 1 |  | 2 |  | 1 | 1 | 1 |
| 18 | Laith J. Abu-Raddad et al, 2021 | 7 (HQ) | 1 | 1 | 1 | 1 |  | 1 |  | 1 | 1 | 0 |
| 19 | Charles F. Schuler et al, 2021 | 5 (MQ) | 0 | 1 | 1 | 1 |  | 1 |  | 1 | 0 | 0 |
| 20 | Chloé Dimeglio et al, 2022 | 5 (MQ) | 0 | 1 | 1 | 1 |  | 0 |  | 1 | 0 | 1 |
| 21 | Laith J. Abu-Raddad et al, 2021 | 8 (HQ) | 1 | 1 | 1 | 1 |  | 1 |  | 1 | 1 | 1 |
| 22 | Hani Abo-Leyah et al, 2021 | 6 (MQ) | 0 | 1 | 1 | 0 |  | 1 |  | 1 | 1 | 1 |
| 23 | Jose Vitale et al, 2021 | 7 (HQ) | 1 | 1 | 1 | 0 |  | 1 |  | 1 | 1 | 1 |
| 24 | Hannah E. Maier et al, 2022 | 7 (HQ) | 1 | 1 | 1 | 0 |  | 1 |  | 1 | 1 | 1 |
| 25 | Sezanur Rahman et al, 2022 | 6 (MQ) | 1 | 1 | 1 | 0 |  | 0 |  | 1 | 1 | 1 |
| 26 | Adrian M. Shields et al, 2021 | 6 (MQ) | 1 | 1 | 1 | 0 |  | 1 |  | 1 | 1 | 0 |
| 27 | Bijaya Kumar Mishra et al, 2021 | 5 (MQ) | 1 | 1 | 1 | 0 |  | 0 |  | 0 | 1 | 1 |
| 28 | Tal Patalon et al, 2022 | 7 (HQ) | 1 | 1 | 1 | 0 |  | 1 |  | 1 | 1 | 1 |
| 29 | Luke Muir et al, 2021 | 6 (MQ) | 0 | 1 | 1 | 0 |  | 1 |  | 1 | 1 | 1 |
| 30 | Michael B. Rothberg et al, 2022 | 8 (HQ) | 1 | 1 | 1 | 1 |  | 1 |  | 1 | 1 | 1 |
| 31 | Kevin B Spicer et al, 2022 | 6 (MQ) | 0 | 1 | 1 | 0 |  | 1 |  | 1 | 1 | 1 |
| 32 | Peter Nordstrom et al, 2022 | 9 (HQ) | 1 | 1 | 1 | 1 |  | 2 |  | 1 | 1 | 1 |
| 33 | Lior Rennert et al, 2022 | 5 (MQ) | 0 | 1 | 1 | 0 |  | 1 |  | 1 | 0 | 1 |
| 34 | Mattia Manica et al, 2021 | 8 (HQ) | 1 | 1 | 1 | 1 |  | 1 |  | 1 | 1 | 1 |
| 35 | Stefan Pilz et al, 2021 | 6 (MQ) | 1 | 1 | 1 | 0 |  | 0 |  | 1 | 1 | 1 |
| 36 | John T. Wilkins et al, 2022 | 6 (MQ) | 0 | 1 | 1 | 0 |  | 1 |  | 1 | 1 | 1 |
| 37 | Baharak Babouee Flury  et al, 2022 | 6 (MQ) |  |  |  |  |  |  |  |  |  |  |
| 37 | Baharak Babouee Flury  et al, 2022 | 6 (MQ) | 0 | 1 | 1 | 0 |  | 1 |  | 1 | 1 | 1 |
| 38 | Priscilla Kim  et al, 2022 | 6 (MQ) | 0 | 1 | 1 | 0 |  | 1 |  | 1 | 1 | 1 |
| 38 | Priscilla Kim  et al, 2022 | 6 (MQ) | 1 | 1 | 1 | 0 |  | 0 |  | 1 | 1 | 1 |
| 39 | Tal Patalon  et al, 2023 | 7 (HQ) | 1 | 1 | 1 | 0 |  | 0 |  | 1 | 1 | 1 |
| 40 | Michael B Rothberg  et al, 2023 | 6 (MQ) | 0 | 1 | 1 | 0 |  | 2 |  | 1 | 1 | 1 |
| 40 | Michael B Rothberg  et al, 2023 | 6 (MQ) | 1 | 1 | 1 | 0 |  | 0 |  | 1 | 1 | 1 |
| 40 | Michael B Rothberg  et al, 2023 | 6 (MQ) | 1 | 1 | 1 | 0 |  | 0 |  | 1 | 1 | 1 |

0: No; 1: Yes. LQ: Low quality; MQ: Moderate quality; HQ: High quality.

# A score of 0–3 solid stars was considered as a LQ study, a score of 4–6 solid stars was considered as a MQ study, and a score of 7–9 solid stars was considered as a HQ study.

# Supplementary Figure 1. Raw forest plot of the pooled incidence rate ratio for SARS-CoV-2 infection comparing baseline seropositive and seronegative individuals.


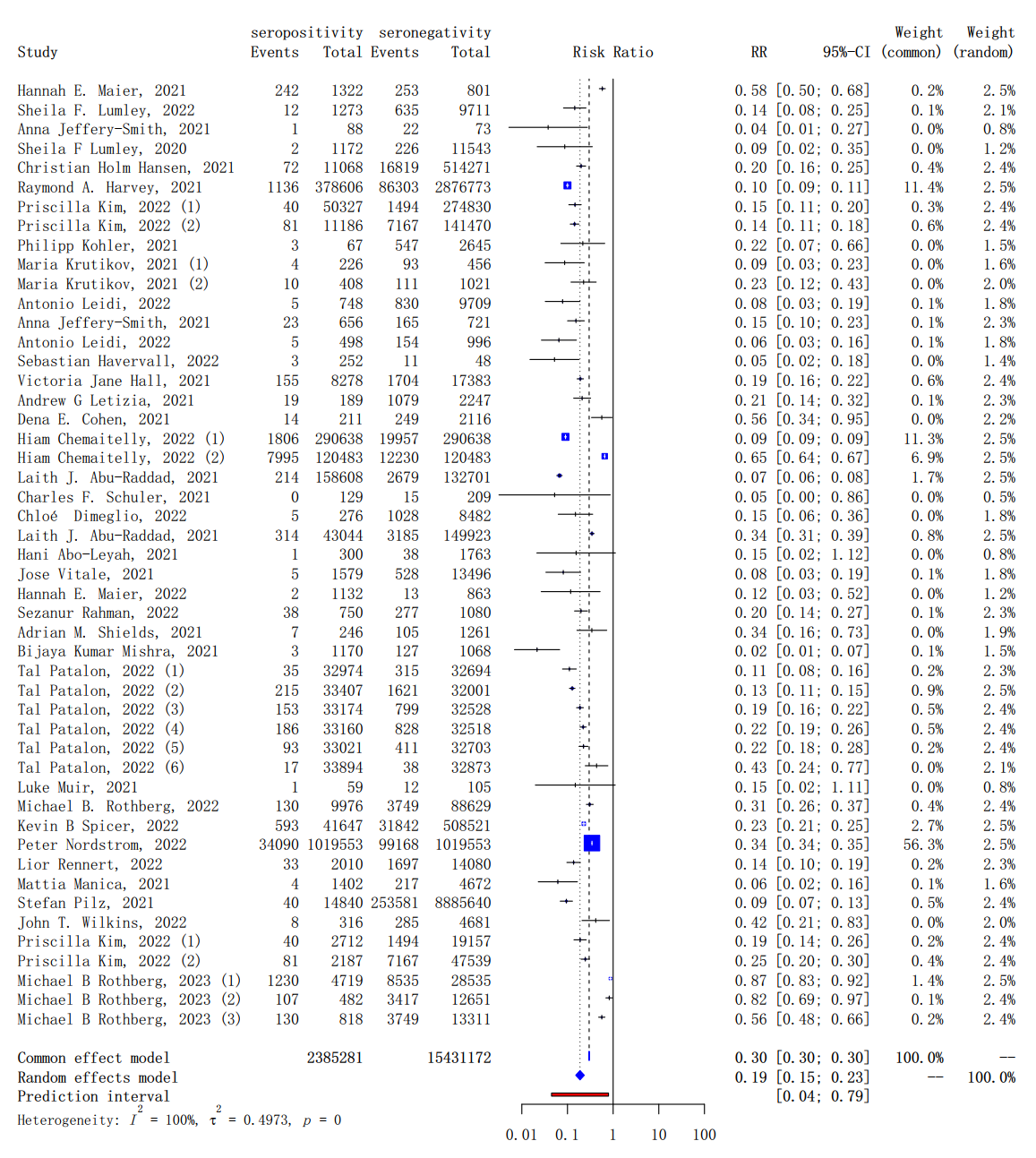


# Supplementary Figure 2. Sensitivity analysis for pooled incidence rate ratio


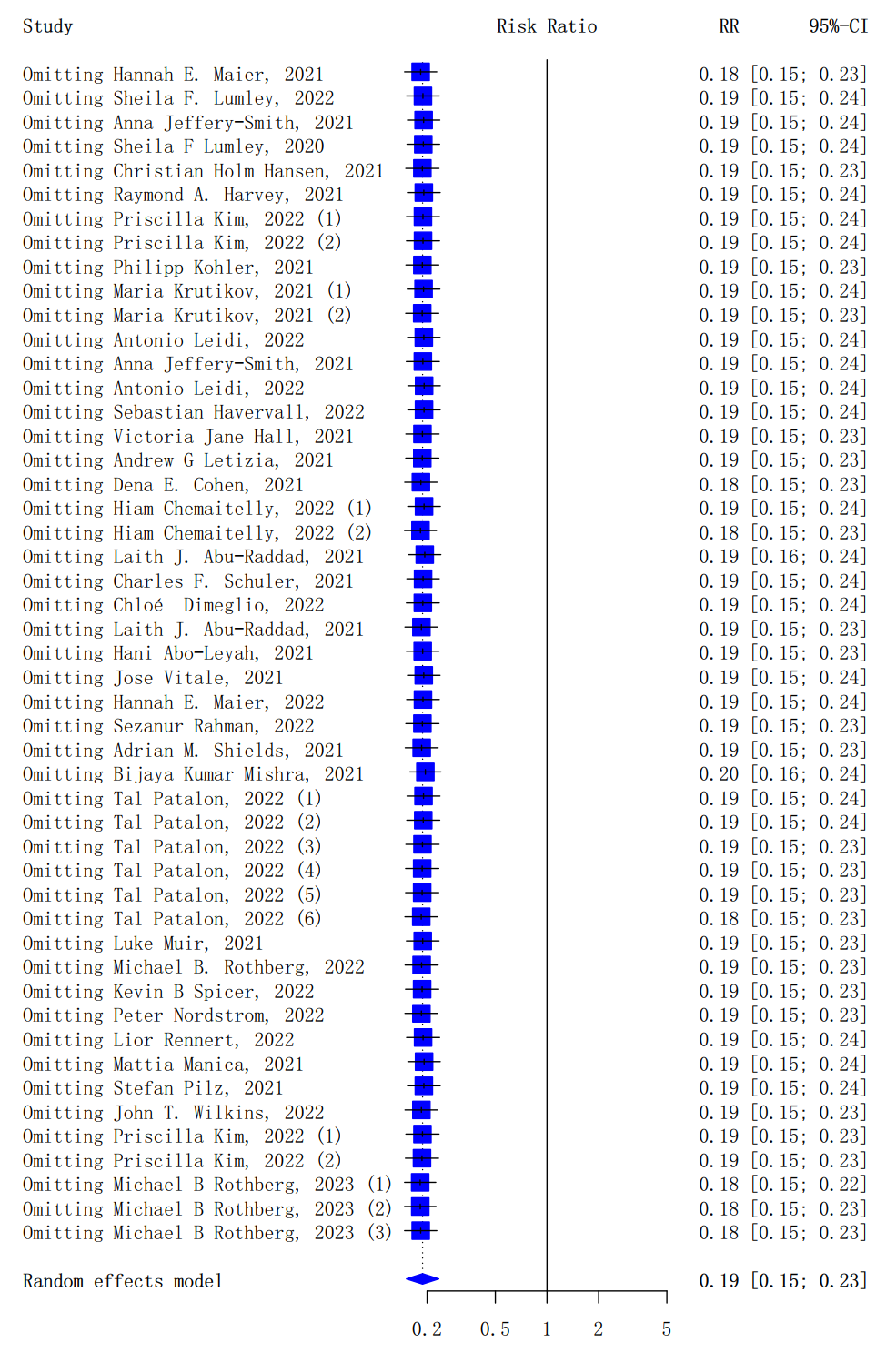


# Supplementary Figure 3. Funnel plot for publication bias before and after trimming and filling

**
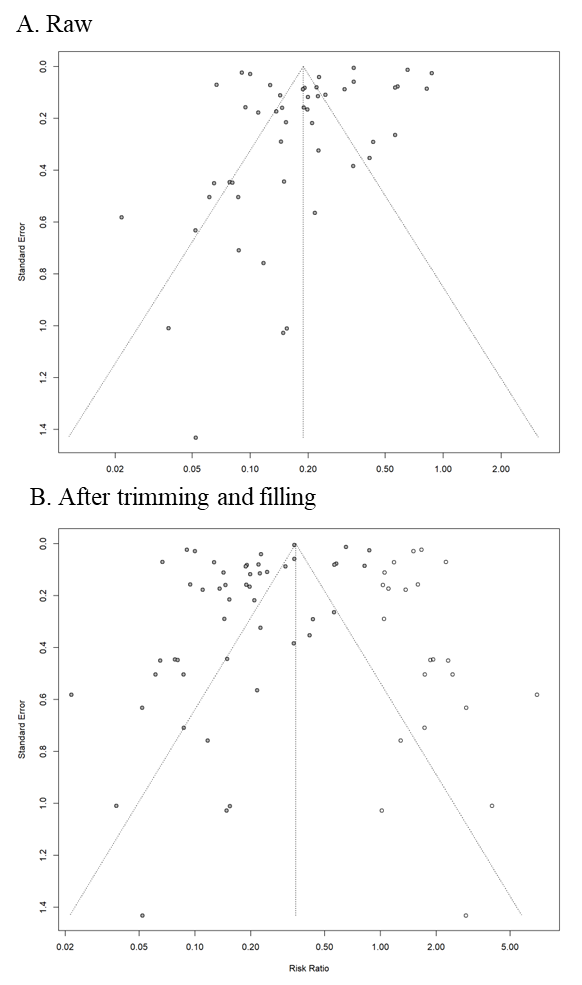
**

# Supplementary Figure 4. Forest plot of the pooled incidence rate ratio for SARS-CoV-2 infection comparing baseline seropositive with seronegative individuals in general population subgroup, HCWs subgroup and hemodialysis patient subgroup

**
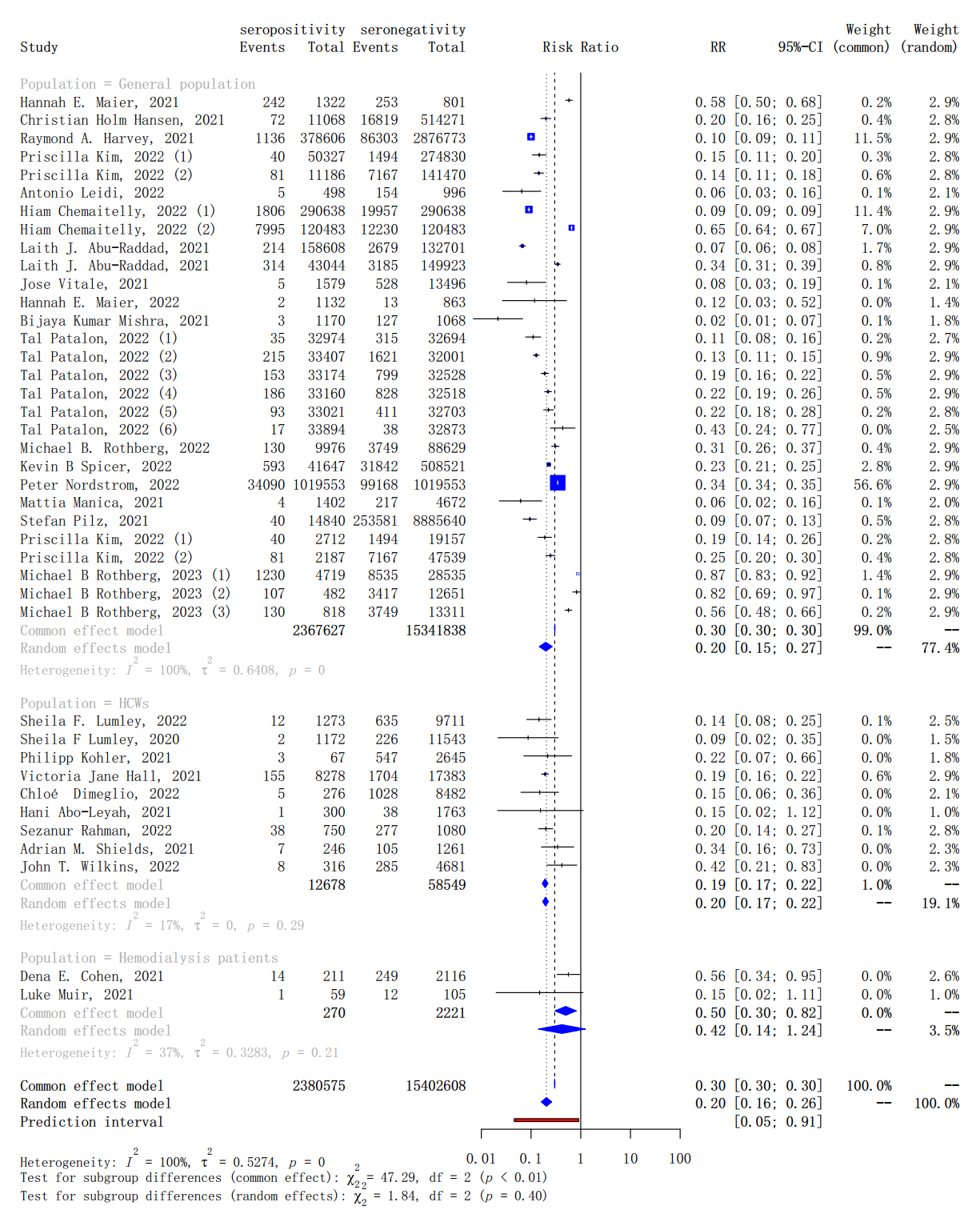
**

# Supplementary Figure 5-1. Forest plot of the pooled incidence rate ratio for SARS-CoV-2 infection comparing baseline seropositive with seronegative individuals in <60 years old subgroup and ≥60 years old subgroup


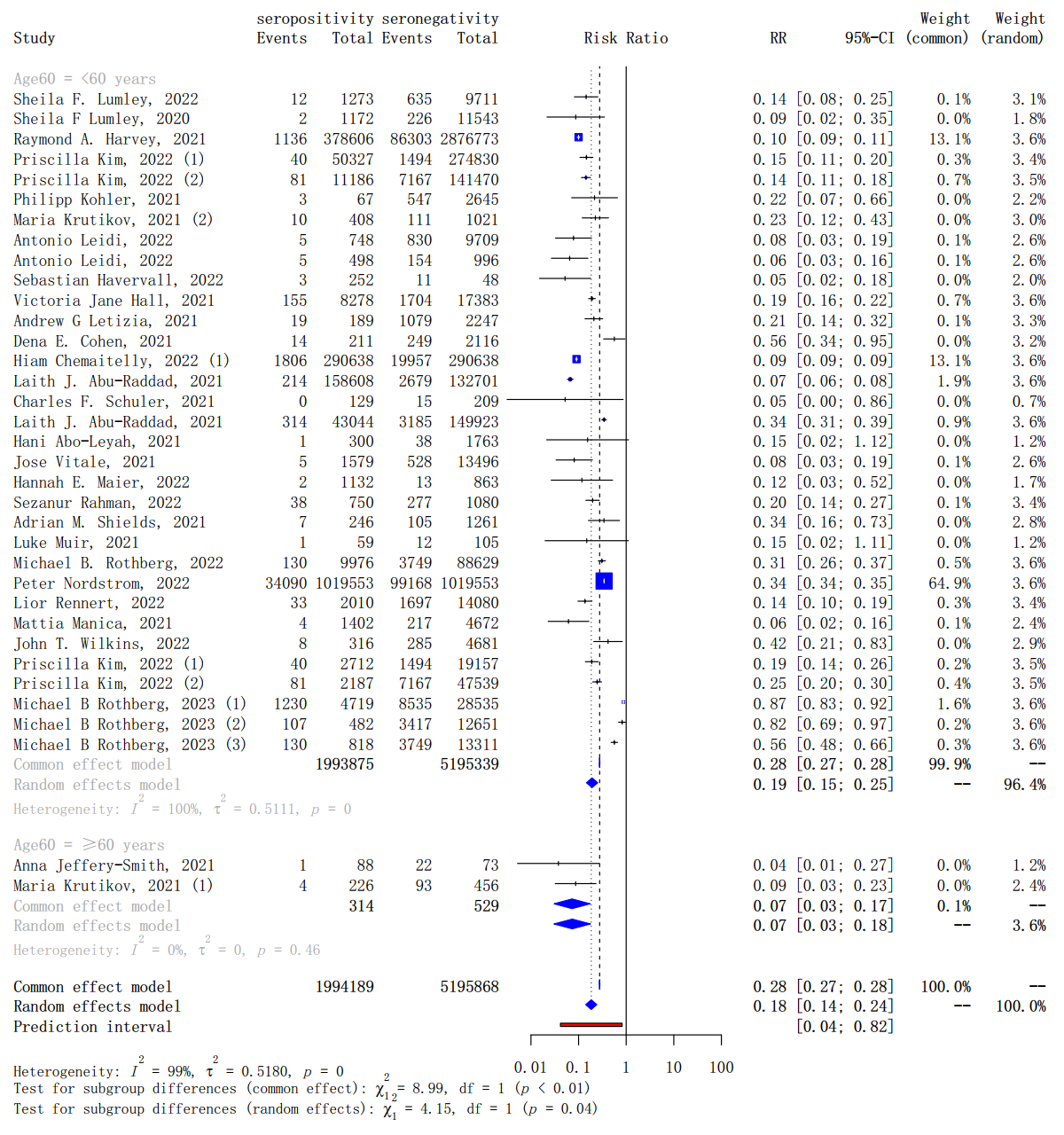


# Supplementary Figure 5-2. Forest plot of the pooled incidence rate ratio for SARS-CoV-2 infection comparing baseline seropositive with seronegative individuals in <55 years old subgroup and ≥55 years old subgroup

**
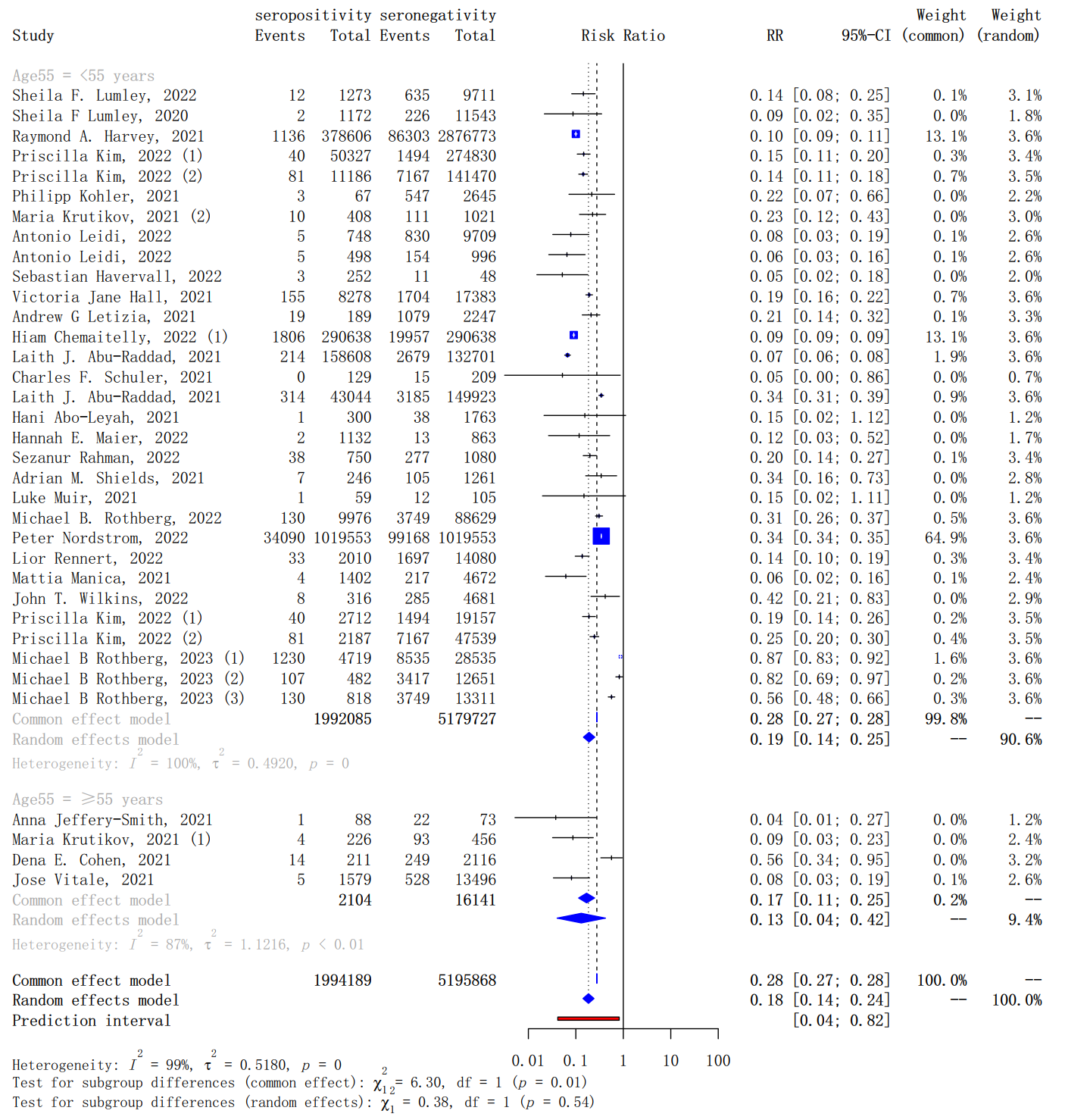
**

# Supplementary Figure 6. Forest plot of the pooled incidence rate ratio for SARS-CoV-2 infection comparing baseline seropositive with seronegative individuals in subgroup of different countries

**
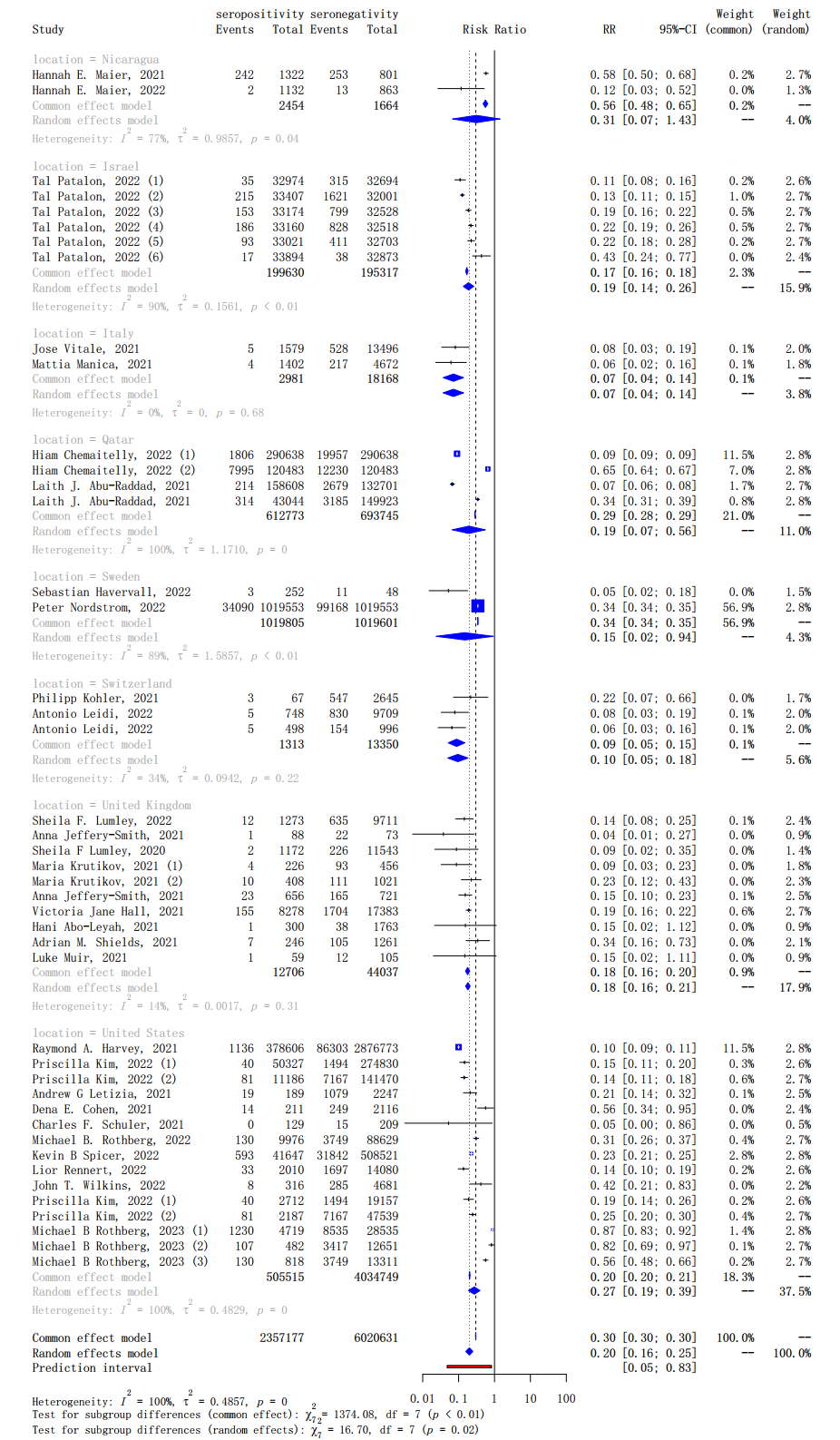
**

#

# Supplementary Figure 7. Forest plot of the pooled incidence rate ratio for SARS-CoV-2 infection comparing baseline seropositive with seronegative individuals in moderate-quality and high-quality group


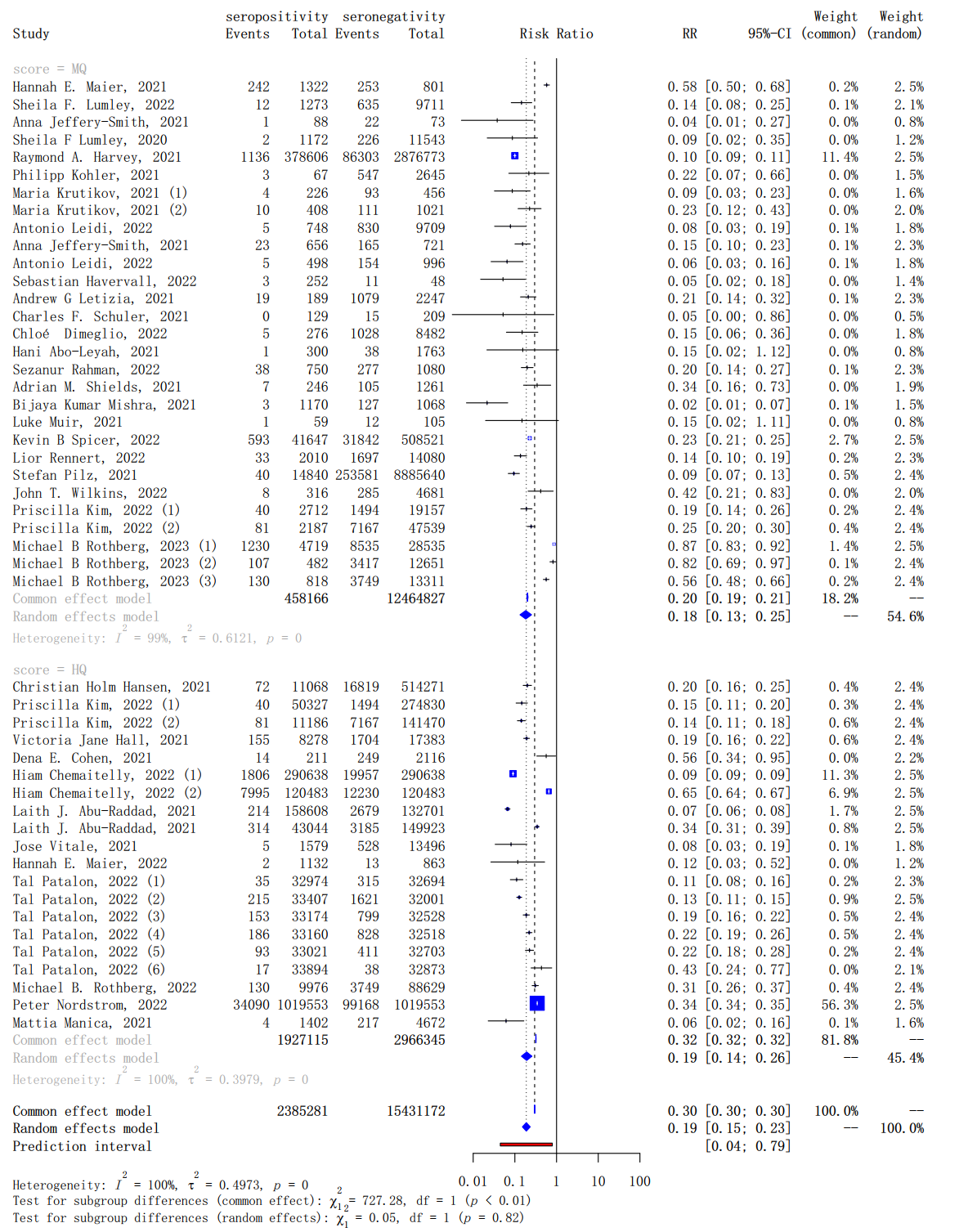


# Supplementary Figure 8-1. Forest plot of the pooled incidence rate ratio for SARS-CoV-2 infection comparing baseline seropositive with seronegative individuals in subgroup of different publication year


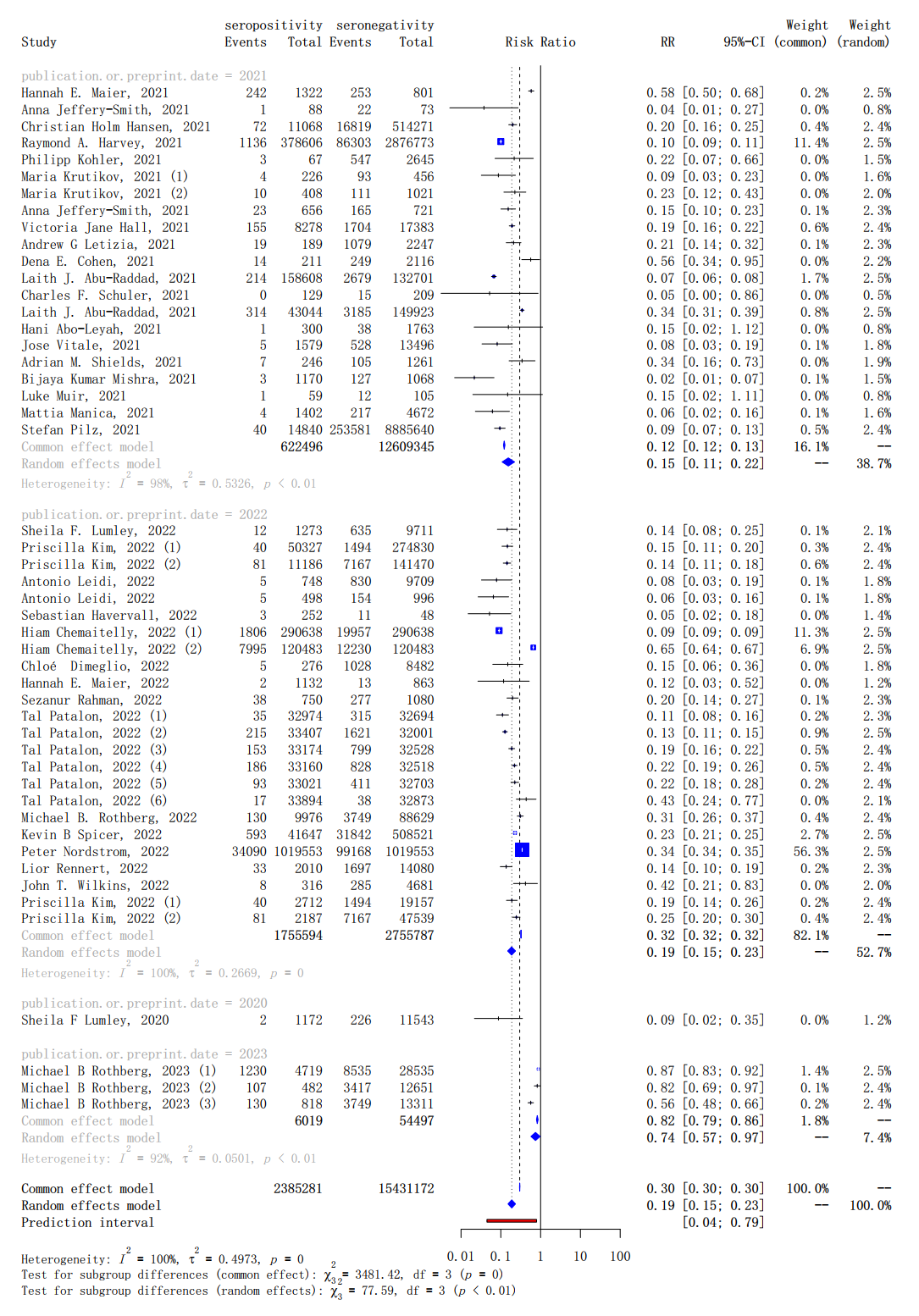


#

# Supplementary Figure 8-2. Forest plot of the pooled incidence rate ratio for SARS-CoV-2 infection comparing baseline seropositive with seronegative individuals in subgroups of inclusion end time of population


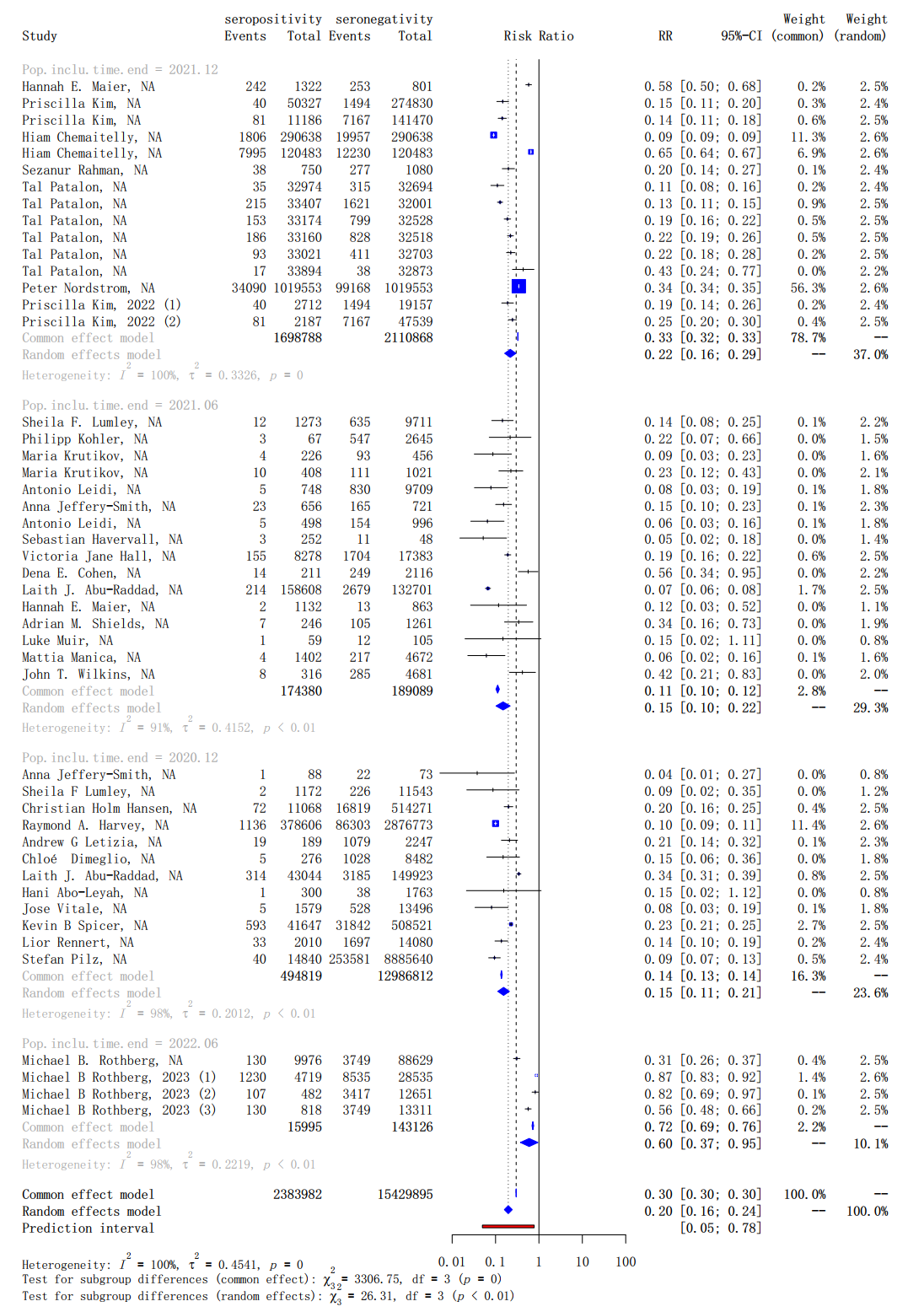


# Supplementary Figure 9. The changing trend of incidence rate ratio after prior infection

**
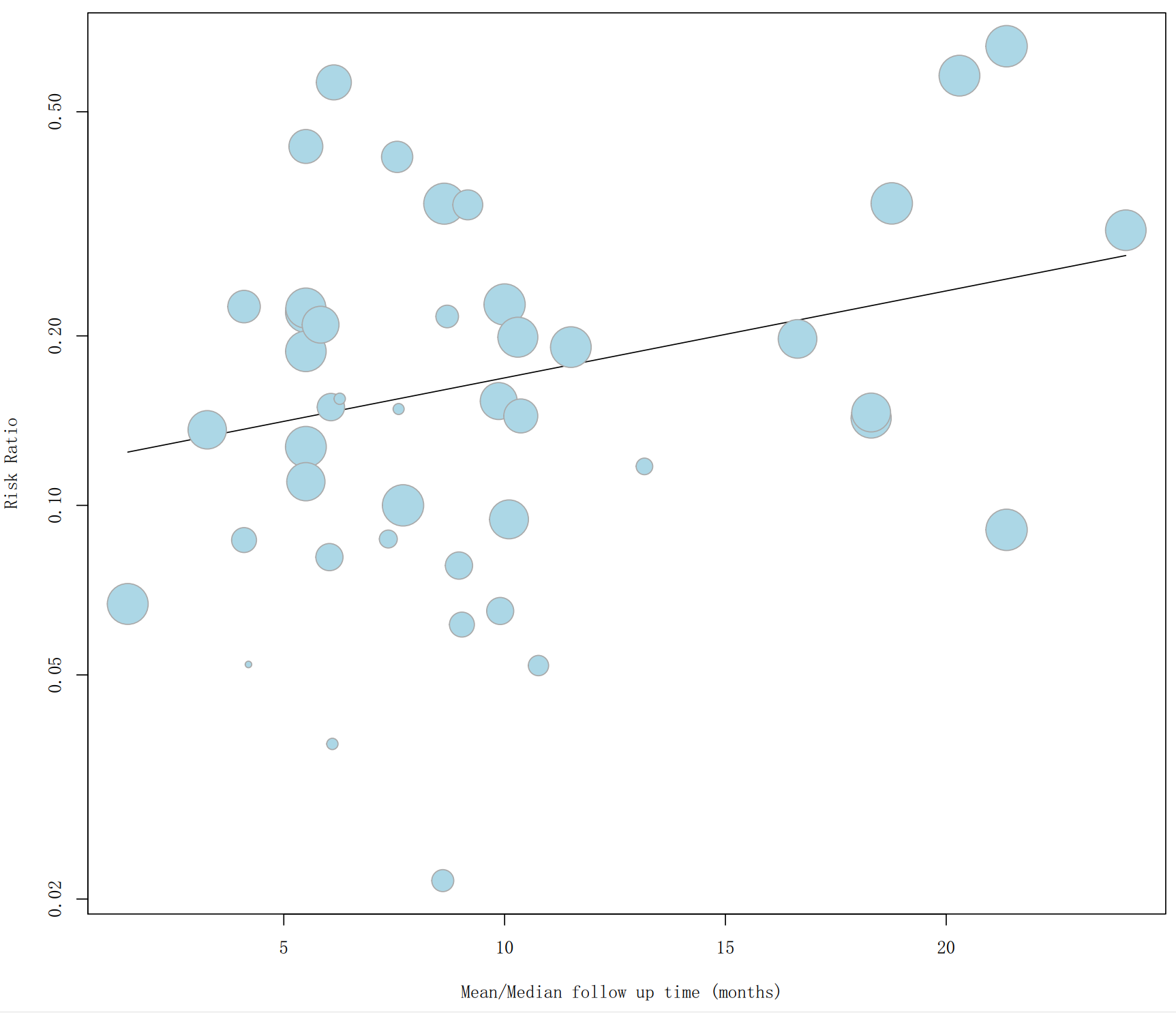
**

# Supplementary Figure 10. Forest plot of the pooled incidence rate ratio for SARS-CoV-2 infection comparing baseline seropositive with seronegative individuals in subgroup of different definitions of reinfection
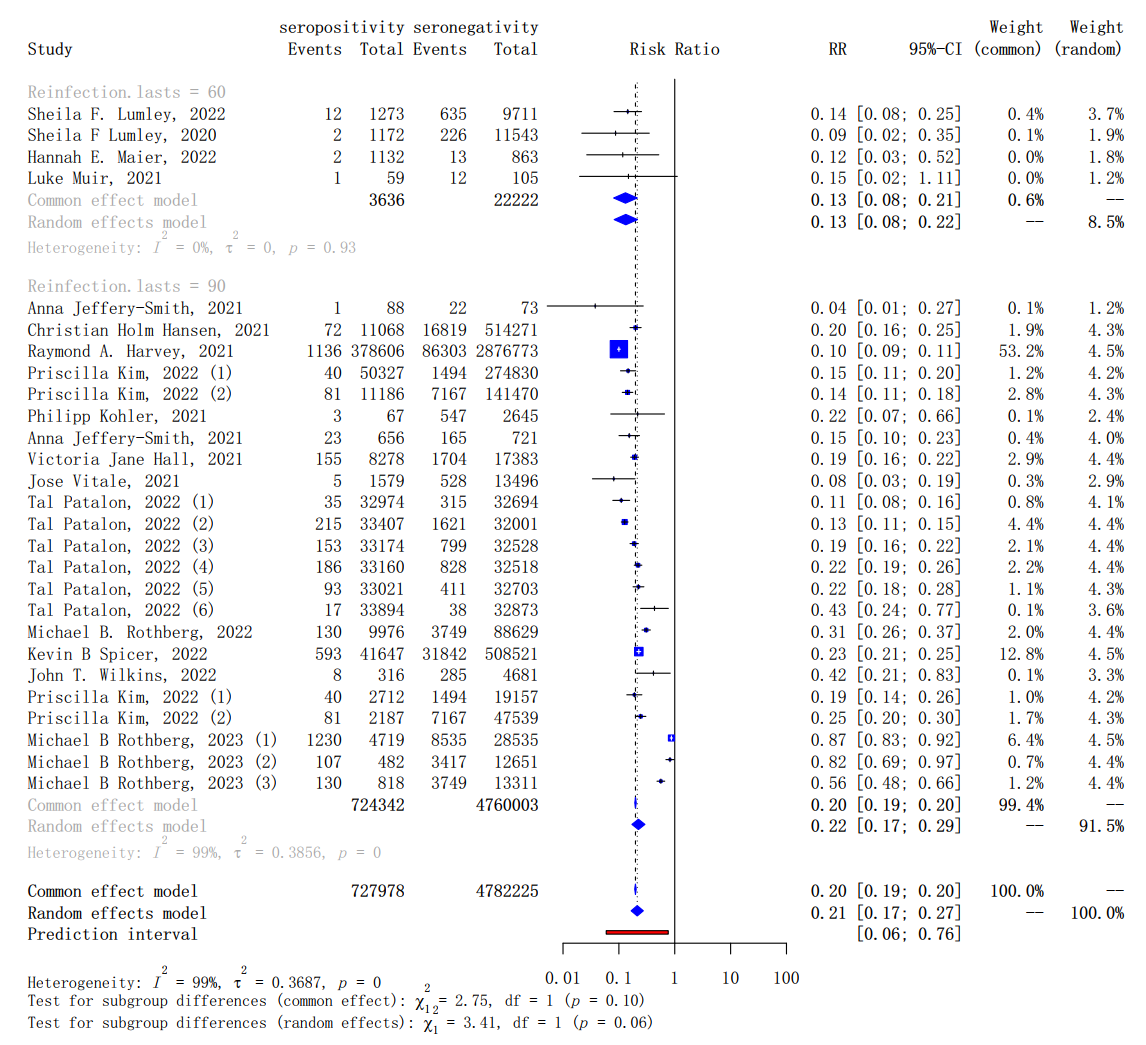

Supplement: Supplementary file 1 [file Data_Sheet_1.docx]
